# Weighted Joint Sentiment-Topic Model for Sentiment Analysis Compared to ALGA: Adaptive Lexicon Learning Using Genetic Algorithm

**DOI:** 10.1155/2022/7612276

**Published:** 2022-07-31

**Authors:** Amjad Osmani, Jamshid Bagherzadeh Mohasefi

**Affiliations:** ^1^Department of Computer Engineering, Qazvin Branch, Islamic Azad University, Qazvin, Iran; ^2^Department of Computer Engineering, Urmia Branch, Islamic Azad University, Urmia, Iran; ^3^Department of Computer Engineering, Urmia University, Urmia, Iran

## Abstract

Latent Dirichlet Allocation (LDA) is an approach to unsupervised learning that aims to investigate the semantics among words in a document as well as the influence of a subject on a word. As an LDA-based model, Joint Sentiment-Topic (JST) examines the impact of topics and emotions on words. The emotion parameter is insufficient, and additional parameters may play valuable roles in achieving better performance. In this study, two new topic models, Weighted Joint Sentiment-Topic (WJST) and Weighted Joint Sentiment-Topic 1 (WJST1), have been presented to extend and improve JST through two new parameters that can generate a sentiment dictionary. In the proposed methods, each word in a document affects its neighbors, and different words in the document may be affected simultaneously by several neighbor words. Therefore, proposed models consider the effect of words on each other, which, from our view, is an important factor and can increase the performance of baseline methods. Regarding evaluation results, the new parameters have an immense effect on model accuracy. While not requiring labeled data, the proposed methods are more accurate than discriminative models such as SVM and logistic regression in accordance with evaluation results. The proposed methods are simple with a low number of parameters. While providing a broad perception of connections between different words in documents of a single collection (single-domain) or multiple collections (multidomain), the proposed methods have prepared solutions for two different situations (single-domain and multidomain). WJST is suitable for multidomain datasets, and WJST1 is a version of WJST which is suitable for single-domain datasets. While being able to detect emotion at the level of the document, the proposed models improve the evaluation outcomes of the baseline approaches. Thirteen datasets with different sizes have been used in implementations. In this study, perplexity, opinion mining at the level of the document, and topic_coherency are employed for assessment. Also, a statistical test called Friedman test is used to check whether the results of the proposed models are statistically different from the results of other algorithms. As can be seen from results, the accuracy of proposed methods is above 80% for most of the datasets. WJST1 achieves the highest accuracy on Movie dataset with 97 percent, and WJST achieves the highest accuracy on Electronic dataset with 86 percent. The proposed models obtain better results compared to Adaptive Lexicon learning using Genetic Algorithm (ALGA), which employs an evolutionary approach to make an emotion dictionary. Results show that the proposed methods perform better with different topic number settings, especially for WJST1 with 97% accuracy at |*Z*| = 5 on the Movie dataset.

## 1. Introduction

Opinion extraction is one of the main branches of natural language processing (NLP) research. Comment extraction (emotion analysis) now is widely used in websites containing different types of merchandise. Online product reviews can help customers buy a product and help manufacturers discover new opportunities by analyzing user feedback. Consequently, automated analysis of reviews is critical. Emotion Analyzer can browse comments on the web and categorize many comments as positive or negative tags. This research is important because it makes managing customer requests easier and more efficient because product owners automatically extract customer feedback and use customer feedback to sell products. There are different methods for extracting opinions and analyzing them, and in this research, an intelligent method has been used [1–7]. Topic modeling presumes that the input text document set contains several unknown subjects that need recognition. Each subject (topic) is an unknown distribution of words, and each review (text document) is a distribution of subjects. The aim is to detect concealed knowledge in textual data related to the user's comments. Several methods perform subject modelings, such as Latent Dirichlet Allocation (LDA) and Probabilistic Latent Semantics Analysis (PLSA). PLSA is a method that can produce the data perceived in a document-term matrix. LDA is a probabilistic method because it is exhibited in a probabilistic language, and it is a generative model because it is about ensuring that documents are produced. LDA has based on the premise that a review is a combination of subjects in which each topic is distributed over words. The linear growth of PLSA parameters indicates that the method is prone to overfitting. LDA can be easily extended to new documents. In addition, increasing the training data size does not lead to the growth of LDA-related parameters [7].

In LDA, subjects are related to documents, and words are related to subjects. To model the emotion of reviews, Joint Sentiment-Topic (JST) [8] establishes an extra layer of emotion between the layers of document and subject, where the emotion labels are related to the documents, the subjects are related to the emotion labels, and words are tagged with emotions and related topics. This study assumes that each word in a document affects its neighbors, and different words in the document may be affected simultaneously by several neighbor words. Thus, the proposed models consider the effect of words on each other. The proposed models add two parameters (weight and window) to JST. The window parameter represents the range of the effect of a word, and the weight parameter represents the strength of the effect of the word. These two parameters play an important role in better classification, as seen in the evaluation section. Using the parameters weight and window, two new methods are introduced that have revealed notable dominance over the baseline algorithms, such as JST, Topic Sentiment modeling (TS) [9], Reverse-JST (RJST) [10], and Tying-JST model (TJST) [8].

More and more improved algorithms and strategies are used to solve sentiment analysis problems. However, none of the researchers have improved the accuracy besides generating a sentiment dictionary. Different from other related studies, in this study, the proposed models improve topic-model-based sentiment classification using two parameters (weight and window). The proposed models consider the effect of words on each other. They can also generate a sentiment dictionary that includes words and scores that specify positive and negative labels and their weight. Accuracy is calculated using two formulas. Finally, by evaluating the proposed methods and the comparison with other algorithms on thirteen datasets of different sizes, the results show that the algorithms presented in this study are superior to the compared algorithms in terms of accuracy, perplexity, and topic_coherency.

The rest of this article is arranged as follows: Section 2 shows a summarized overview of previous works in emotion analysis and the use of topic modeling in emotion analysis. The proposed models are provided in Section 3. The evaluation results are discussed in Section 4, and Section 5 concludes this article.

## 2. Related Works

The value of emotion analysis may be highlighted by analyzing customer happiness from online services like email. It is also feasible to employ emotion mining to evaluate the opinions of various people in order to make them aware of things that have favorable reviews. Major types of classification in emotion analysis are document, sentence, and aspect. An opinion is a quadrilateral (*g*, *s*, *h*, *t*), where *g* is the target, *s* is sentiment, *h* is the author's opinion, and *t* is the opinion expression time [11, 12, 13]. Many attempts have been made to detect emotions and explore the knowledge embedded in text data. Topic modeling obtains concealed subjects of documents. In topic modeling, the aim is to discover the best set of hidden variables that can express the observed data. LDA has been used as a topic model to effectively explore subjects in the documents [7]. LDA has motivated countless algorithms to expand to solve different problems [14–17]. In [18], the authors exhibit three topic models which make better LDA using date, helpfulness, and subtopic parameters. Articles [8, 10, 19] describe the methodology JST. This model expands LDA using a sentiment layer. This method cannot accurately identify the different emotions and is used as a baseline method in most articles. Several methods are similar to JST [8, 10, 20]). The aspect and Sentiment Unification Model (ASUM) [20] is similar to JST. JST assumes that each word represents an aspect, but ASUM assumes that each sentence represents a description of an aspect. A variation of the JST model is TJST [8]. The main difference between JST and TJST is that to sample a word in a document during the generative process of documents, JST selects a subject-document distribution for each document, whereas TJST uses one subject-document distribution for all documents. According to [10], the emotion influences the subject in JST, whereas in RJST, the subject influences the emotion. According to [9], there is only one topic-sentiment distribution for all documents in the TS, while there is a distribution for each document in RJST.

Several methods have been introduced for text emotion analysis that uses topic modeling [21–23, 78]. In [24], the authors introduce an algorithm that creates a review containing both shared subjects and subjects distributed over words as special data. Two topic models are proposed in [79]: Multilabel Supervised Topic Model (MSTM) and Sentiment Latent Topic Model (SLTM). Both methods could be used to categorize social emotions. In [25], the authors introduce a Sentiment Enriched and Latent-Dirichlet-Allocation-based review rating Prediction (SELDAP) to predict ratings using topics and sentiments of reviews. In [26], the authors introduce a method named Hierarchical Clinical Embeddings combined with Topic modeling (HCET), which can integrate five types of Electronic Health Record (EHR) data over several visits to predict depression. The authors of [80] presented the word Sense aware LDA (SLDA) approach that uses word sense in topic formation. In [27], the authors introduce a survey of different short text topic modeling methods. They provide a detailed analysis of algorithms and discuss their performance. The authors proposed a segment-level joint topic-sentiment model (STSM) in [81], where each sentence is divided into parts by conjunctions, and the assumption that all terms in a section convey the same emotion is presented. In [28], the authors provided a thorough examination of subject modeling methods. 

Deep learning provides an approach to utilizing large volumes of calculation and data using little manual engineering. Recently, deep learning approaches to analyzing emotions have reached a considerable triumph [29, 30, 47, 77]. Optimization methods have developed significantly in recent years [31–37]. Optimization methods are widely used in the feature section, notably for text. In [38], the authors proposed a multiobjective-grey wolf-optimization algorithm to categorize sentiments. In [39], the authors proposed a binary grey wolf optimizer method to classify labels in the text. In the following article [40], the authors introduced a new optimization method that mimics the model of a successful person in society. Their article used this method to categorize emotions, which achieved very good results. There are several works on using user behavior for sentiment analysis. Tag sentiment aspect (TSA) framework, a new probabilistic generative topic framework, was presented by [48] with three implementation editions. TSA is on the basis of LDA. In [41], the authors concentrate on user-based methods on social networks, where users create text data to show their views on different topics and make connections with other users to create a social network. In [42], the authors used a signed social network to detect the emotions of reviews as an unsupervised approach. Various works use other techniques for sentiment analysis problems [43–45]. In Adaptive Lexicon learning using a Genetic Algorithm (ALGA) [46], some emotion dictionaries for a dataset in the training stage are constructed using the genetic method. These sets are utilized in the testing stage. Each lexicon comprises both words and their scores. A chromosome is modeled as a vector of emotional words and scores in the genetic approach. Scores are in the range of (the lowest score of an emotional word, the highest score of an emotional word). The main goal of ALGA is to create a lexicon that minimizes the error in the training stage.

In [47], the authors proposed a deep learning-based topic-level opinion mining method. The approach is novel in that it works at thelevel of the sentence to explore the subject using online latent semanticindexing and then employs a subject-level attention method in an extendedshort-term memory network to detect emotion. In [62], the authors proposed a joint aspect-based sentiment topic model that extracts multigrained aspects and emotions. In [49], parts-of-speech (POS) tagging is performed via a hidden Markov model, and unigrams, bigrams, and bi-tagged features are extracted. Also, the nonparametric hierarchical Dirichlet process is employed to extract the joint sentiment-topic features. In [50], the authors used an unsupervised machine learning method to extract emotion at the document and word levels. In [51], the authors proposed a new framework for joint sentiment-topic modeling based on the Restricted Boltzmann Machine (RBM), a type of neural network. In [52], the authors proposed a probabilistic method to incorporate textual reviews and overall ratings, considering their natural connection for a joint sentiment-topic prediction. In [53], the authors proposed a hybrid topic model-based method for aspect extraction and emotion categorization of reviews. LDA is used for aspect extraction and two-layer bidirectional long short-term memory for emotion categorization. In [54], the authors proposed a joint sentiment-topic model that uses Markov Random Field Regularizer and can extract more coherent and diverse topics from short texts. In [55], the authors proposed a topic model with a new document-level latent sentiment variable for each topic, which moderates the word frequency within a topic. In [56], the authors proposed a new method for text emotion detection, aiming to improve the LSTM network by integrating emotional intelligence and attention mechanism. In [57], the authors proposed a new model for aspect-based emotion detection. The model is a novel adaptation of the LDA algorithm for product aspect extraction.

In [58], the authors introduced a new deep learning-based algorithm for emotion detection, using available ratings as weak supervision signals. In [59], the authors introduced a new deep learning-based algorithm for emotion detection, using two hidden layers. The first layer learns sentence vectors to represent the semantics of sentences, and in the second layer, the relations of sentences are encoded. In [60], the authors introduced a transformer-based model for emotion detection that encodes representation from a transformer and applies deep embedding to improve the quality of tweets. In [61], the authors introduced an attention-based deep method using two independent layers. By having to consider temporal information flow in two directions, it will retrieve both past and future contexts.

In this study, the proposed methods have tried to increase the accuracy with fewer parameters and, at the same time, simplicity compared to the existing methods. The proposed methods analyze emotions at the document-level and create an emotional dictionary. They are also the first methods that create an emotional dictionary through a topic modeling technique automatically and accurately. The proposed methods are the first methods that consider the words in the text and their effect on each other in a dynamic and weighty way.

Table 1 compares a number of articles presented in recent years in emotion analysis in terms of method, language, and dataset. In the method column, as can be seen, the combination of topic modeling and deep learning methods has recently been considered. In the language column, it is specified in which language the proposed method has been tested. The name of the dataset that has been tested can also be seen in the dataset column.

## 3. Proposed Models

This study proposes two novel topic sentiment models called Weighted Joint Sentiment-Topic (WJST) and Weighted Joint Sentiment-Topic 1 (WJST1). The proposed models improve JST using two extra parameters (weight and window).

**Figure 1:**
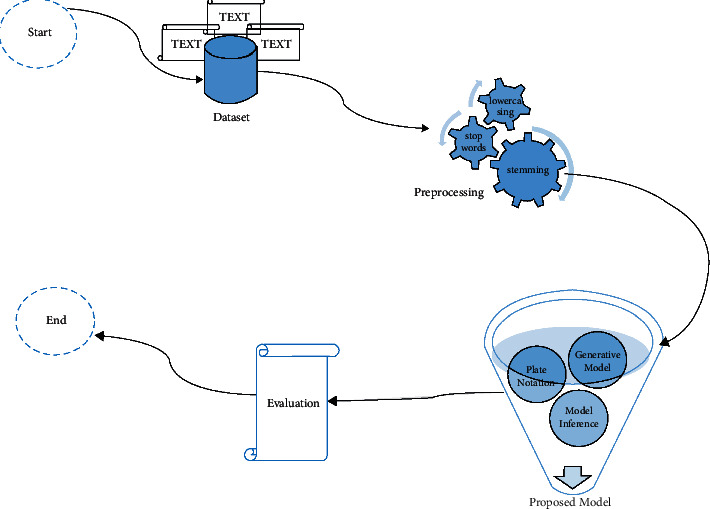
The framework chart of the proposed methods.

According to Figure 1, the data type of the **dataset** is text. **Preprocessing** is performed by lowercasing all words, removing the stop words and words with too low and too high frequency, stemming, removing digits, and removing nonalphabetic characters such as (#, ! …). Proposed models can be summarized as follows: (1) in the **Generative Model** part, the procedure of generating a word in a document under a topic model is illustrated. (2) In the **Plate Notation** part, a graphical representation of the subject model is provided (in the style of plate notation). (3) In the **Model Inference** part, Gibbs sampling will be used (to fulfill approximation inference). In the **Evaluation** phase, the model's performance is evaluated using accuracy, perplexity, and topic_coherency.

### 3.1. Motivation

The proposed models add two parameters to JST as latent variables in this study. From our view, it is assumed that the words in the documents affect their neighbors, and different words in the document may be affected simultaneously by several neighbor words. For example, in the sentence “My phone has a small memory, and its pictures quality is low,” the unigram *small* affects the unigram *memory*, and the bigram *small memory* affects the unigrams *phone* and *pictures*. So, unigram *small* affects unigrams *memory*, *phone*, and *picture*.

**Figure 2:**
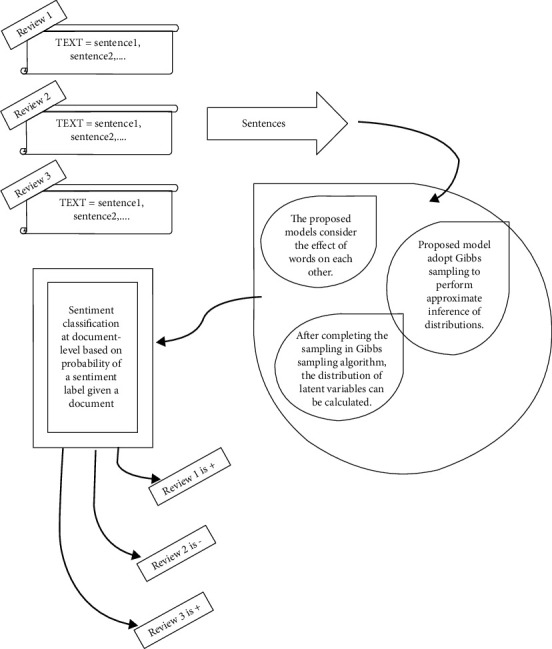
The general architecture of the proposed methods.

According to Figure 2, the reviews as input text data types are used for sentiment classification. The proposed models consider the effect of words on each other. They adopt Gibbs sampling to perform approximate inference of distributions. After completing the sampling in the Gibbs sampling algorithm, latent variables' distribution can be calculated. Sentiment classification at the document-level is calculated based on the probability of a sentiment label given to a document.

**Figure 3:**
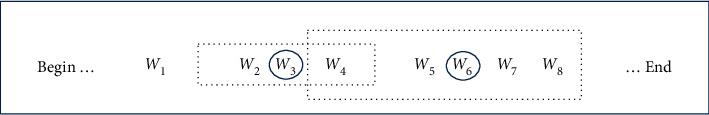
An example of a sentence with different windows.

Like the above example, a word can affect neighbor words in many sentences. So, in the proposed models, we consider the effect of words on each other using two parameters. The window parameter represents the range of the impact of a word, and the weight parameter represents the strength of the effect of the word. In the proposed models, each word has a weight, a sentiment label, and a topic and affects its neighbors as much as its window size, which means that each word has a window. For instance, as can be seen in Figure 3, word *w*_3_ has the window size equal to 1 and affects words *w*_2_ and *w*_4_, and *w*_6_ has the window size equal to 2 and affects words *w*_4_, *w*_5_,  *w*_7_, and *w*_8_. If word *w*_3_ had weight *h* and negative sentiment, words *w*_2_ and *w*_4_ would have weight *h* and negative sentiment as well. Each word is affected by its neighbors. So, different words in a document may be affected simultaneously by several neighbor words.

### 3.2. The Problem Statement

In this study, given a corpus of *|R|* documents, *R*={*r*_1_, *r*_2_, *r*_3_,…, *r*_|*R*|_}, a document, *r*, consists of {*w*_1_, *w*_2_, *w*_3_,…, *w*_*N*_*r*__} words, and each word belongs to a vocabulary set with a |*V*| distinct element. Furthermore, |*Q|* is the number of separate windows, |*E*| is the number of distinct weights, |*S*| is the number of distinct sentiment labels, and |*Z*| is the number of distinct topics. In the present study, five sets *θ*, *φ*, *π*, *ψ*, and *ξ* require to be inferred which are latent variables. The hyperparameters *α*, *β*, *γ*, *δ*, and *μ* are given based on the experience, which can be the prior observation counts before observing any actual words, where *α* is Dirichlet prior distribution for *θ*, *β* is Dirichlet prior distribution for *φ*, *γ* is Dirichlet prior distribution for *π*, *δ* is Dirichlet prior distribution for *ψ*, and *μ* is Dirichlet prior distribution for *ξ*. The latent parameters **z**, **s**, **q**, **e**, *φ*, *θ*, *π*, *ξ*,  and *ψ* require to be approximated using observed variables, where **z** is topic variable, **s** is sentiment variable, **q** is window variable, and **e** is weight variable. The proposed models demonstrate the process of generating words in documents. Furthermore, they can approximate the latent variables. In the present study, the main aim of the proposed topic models is to categorize sentiments at the document-level.

#### 3.2.1. The Problem We Are Trying to Solve or Improve

Analyzing user satisfaction with various services, products, or movies is the main problem in this study, mainly reflected in users' comments. A user's comment is formed by a message as text on the Internet which can be a tweet or a simple message on a website. So, for example, it is feasible to employ emotion mining to evaluate the opinions of various people in order to make them aware of things that have favorable reviews.

#### 3.2.2. The Solution to the Problem

Many attempts have been made to detect emotions and explore the knowledge embedded in text data. Topic modeling as a known method can obtain concealed subjects of documents. LDA has been used as a topic model to effectively explore issues in the documents. As an LDA-based model, JST examines the impact of topics and emotions on words. The emotion parameter is insufficient, and additional parameters may play valuable roles in achieving better performance.

This study presents two new topic models that extend and improve JST through two new parameters and generate a sentiment dictionary. The proposed models consider the effect of words on each other, which, from our view, is an important factor and can increase the performance of baseline methods. Several methods have been introduced for text emotion analysis that uses topic modeling. However, none of the researchers have improved the accuracy besides generating a sentiment dictionary. Different from other related studies, in this study, the proposed models improve topic-model-based sentiment classification using two parameters (weight and window). The proposed models are deeply described step-by-step in the following sections.

### 3.3. The General Structure of WJST

This subsection introduces a new model named WJST, which improves JST using two parameters (weight and window). The primary goal of WJST is to classify sentiments at the document-level. A summary of symbols applied in the model is prepared in Table 2. The process of generating a word of a document in WJST can be outlined as follows: (1) for each document, an author first decides the distribution of sentiments. For example, sentiments are 70% positive and 30% negative, so the proposed model chooses a sentiment label from the per-document sentiment distribution. (2) After determining the sentiment label, the author writes a review about a product according to the distribution of topics. For example, topics are 70% about memory, 20% about speed, and 10% about battery, so WJST chooses a topic from the per-document topic distribution that depends on the sentiment label. (3) After determining the sentiment label and the topic, the author decides the distribution of weights and the distribution of windows. WJST then chooses a weight from the per-document weight distribution that depends on the sentiment label and topic. WJST chooses a window size from the per-document window distribution that depends on the topic. (4) Finally, the author chooses some words to express an opinion under the identified topic, sentiment label, weight, and window. So, WJST draws a word from the per-corpus word distribution that depends on the topic, sentiment label, weight, and window. The words with different topics may have different window sizes. For example, a word with topic *memory* has a smaller window size than a word with topic *mobile* because topic *mobile* is more general than topic *memory* which can cover topic *memory*. So, the **topic affects window size**.

**Figure 4:**
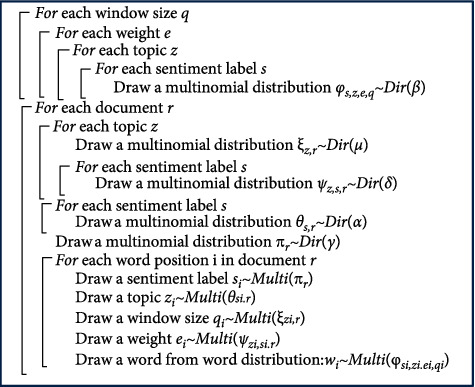
The formal definition of the process of generating words in WJST.

The words with different topics may have different weights. For example, word *size* in topic *memory* is significant and considerable weight because all customers like memories with larger capacity. Word *size* in topic *mobile* is not important as word *size* in topic *memory,* and it has a small weight in topic *mobile* because some customers may like mobile phones with small size (iPhone 6s), and some customers may like the mobile phones with large size (iPhone 6s+). So, the **topic affects weight**. The words with different sentiment labels may have different weights. For example, suppose that topic *memory* contains two words *size* and *cost*. If the word *size* is positive, positive size will be more important than the word *cost,* and its weight will be larger than the cost. If word *size* is negative, the positive cost will be more important than the word *size,* and its weight will be larger than the size. Positive size means using words like *large* and *big* because customers like memories with larger capacity sizes. Negative size means using words like *small* because costumers do not like memories with smaller capacity size. Positive cost means using words like *low* and *cheap* because costumers like low-priced memories. Negative cost means using words like *high* and *expensive* because costumers do not like high-priced memories. So, **sentiment label affects weight**. The proposed model is parametric in this study [63]. Furthermore, the number of topics is constant. The generative model of WJST is demonstrated in Figure 4.

**Figure 5:**
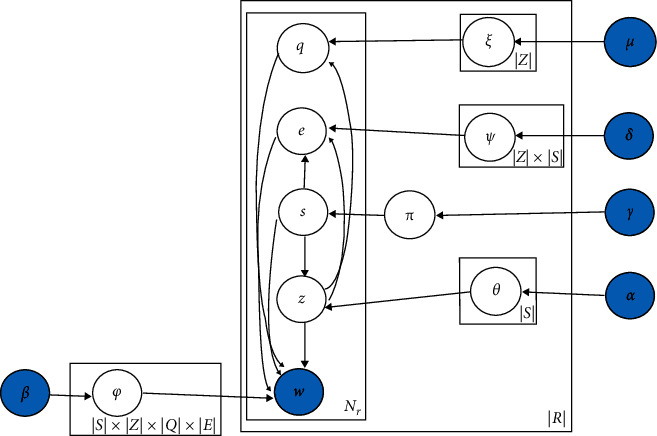
The plate notation of WJST.

The symbols of *Multi* and *Dir* demonstrate distributions of Multinomial and Dirichlet, respectively. Five sets of latent variables *θ*, *φ*, *π*, *ψ*,  and *ξ* require to be inferred which are latent variables. The hyperparameters *α*, *β*, *γ*, *δ*, and *μ* are given based on the experience, which can be the prior observation counts before observing any actual words. The latent parameters **z**, **s**, **q**, **e**, *θ*, *φ*, *π*, *ψ*,  and *ξ* require to be approximated using observed variables. The plate notation of WJST is exhibited in Figure 5. The plate notation is a method for expressing variables repeating in a graphical model. Furthermore, a probabilistic model shows the conditional dependency layout among the random variables as a graph.

According to Figure 5, the joint probability distributions for the model WJST can be factored as follows:

(1) $$\begin{array}{c} P\left(w,z,s,q,e\right)=P\left(w|z,s,q,e\right)\times P\left(z|s,r\right)\times P\left(s|r\right)\times P\left(q|z,r\right)\times P\left(e|z,s,r\right), \end{array}$$

where by integrating out *φ*, we achieve:

(2) $$\begin{array}{c} P\left(w|z,s,q,e\right)={\left(\frac{\Gamma \left(\left|V\right|\times \beta \right)}{\Gamma {\left(\beta \right)}^{\left|V\right|}}\right)}^{\left|Z\right|\times \left|S\right|\times \left|Q\right|\times \left|E\right|}\prod_{z}\prod_{s}\prod_{q}\prod_{e}\frac{\prod_{w}\Gamma \left(N_{w,z,s,q,e}+\beta \right)}{\Gamma \left(N_{z,s,q,e}+\left(\left|V\right|\times \beta \right)\right)}, \end{array}$$

where |V| is the vocabulary size, |*S|* is the number of sentiment labels, |*Z|* is the number of topics, |*Q|* is the number of distinct windows, and |*E|* is the number of weights. The symbol *N*_*w*,*z*,*s*,*q*,*e*_ is the number of times the word *w* has been assigned to topic *z*, window *q*, weight *e*, and sentiment *s*. The symbol *N*_*z*,*s*,*q*,*e*_ is the number of words with topic *z*, window *q*, weight *e*, and sentiment *s*.The symbol *β* is Dirichlet prior to *φ*. The symbol Γ is the gamma function. In addition, by integrating out *θ*, we achieve:

(3) $$\begin{array}{c} P\left(z|s,r\right)={\left(\frac{\Gamma \left(\left|Z\right|\times \alpha \right)}{\Gamma {\left(\alpha \right)}^{\left|Z\right|}}\right)}^{\left|S\right|\times \left|R\right|}\prod_{r}\prod_{s}\frac{\prod_{\text{z}}\Gamma \left(N_{z,s,r}+\alpha \right)}{\Gamma \left(N_{s,r}+\left(\left|Z\right|\times \alpha \right)\right)}, \end{array}$$

where |*R*| is the number of documents and *N*_*z*,*s*,*r*_ is the number of words with topic *z* with sentiment *s* in document *r*. The symbol *N*_*s*,*r*_ is the number of words with sentiment *s* in document *r*. The symbol *α* is Dirichlet before *θ*. And by integrating out *π*, we achieve:

(4) $$\begin{array}{c} P\left(s|r\right)={\left(\frac{\Gamma \left(\left|S\right|\times \gamma \right)}{\Gamma {\left(\gamma \right)}^{\left|S\right|}}\right)}^{\left|R\right|}\prod_{r}\frac{\prod_{\text{s}}\Gamma \left(F_{s,r}+\gamma \right)}{\Gamma \left(F_{r}+\left(\left|S\right|\times \gamma \right)\right)}, \end{array}$$

where *F*_*s*,*r*_ is the effect of words with sentiment *s* in document *r*, which is equal to $\sum_{w\in r}\left|e_{w,s,r}\times \left(1+2\times q_{w,s,r}\right)\right|$ where *e*_*w*,*s*,*r*_ is the weight of word *w* with sentiment *s* in document *r* and *q*_*w*,*s*,*r*_ is the window size of word *w* with sentiment *s* in document *r*. The symbol *F*_*r*_ is the sum of the effect of words with different sentiments (positive and negative) in document *r*, which is equal to ∑_*s*∈{positive, negative}_*F*_*s*,*r*_.The symbol *γ* is Dirichlet before *π*. And by integrating out *ξ*, we achieve:

(5) $$\begin{array}{c} P\left(q|z,r\right)={\left(\frac{\Gamma \left(\left|Q\right|\times \mu \right)}{\Gamma {\left(\mu \right)}^{\left|Q\right|}}\right)}^{\left|Z\right|\times \left|R\right|}\prod_{r}\prod_{z}\frac{\prod_{\text{q}}\Gamma \left(N_{q,z,r}+\mu \right)}{\Gamma \left(N_{z,r}+\left(\left|Q\right|\times \mu \right)\right)}, \end{array}$$

where |*Q|* is the number of distinct windows. The symbol *N*_*q*,*z*,*r*_ is the number of words with topic *z* and window *q* in document *r*. The symbol *N*_*z*,*r*_ is the number of words with topic *z* in document *r*. The symbol *μ* is Dirichlet before *ξ*. And by integrating out *ψ*, we achieve:

(6) $$\begin{array}{c} P\left(e|z,s,r\right)={\left(\frac{\Gamma \left(E|\times \delta \right)}{\Gamma {\left(\delta \right)}^{\left|E\right|}}\right)}^{\left|Z\right|\times \left|S\right|\times \left|R\right|}\prod_{s}\prod_{r}\prod_{z}\frac{\prod_{e}\Gamma \left(N_{e,z,s,r}+\delta \right)}{\Gamma \left(N_{z,s,r}+\left(\left|E\right|\times \delta \right)\right)}, \end{array}$$

where |*E|* is the number of weights, *N*_*e*,*z*,*s*,*r*_ is the number of words with topic *z*, weight *e*, and sentiment *s* in document *r*. The symbol *N*_*z*,*s*,*r*_ is the number of words with sentiment *s* and topic *z* in document *r*. The symbol *δ* is Dirichlet before *ψ*. To estimate the parameters *φ*, *θ*, *π*, *ξ*,  and *ψ*, we need to evaluate the above distributions. These distributions are difficult to assess directly, so we adopt Gibbs sampling to perform approximate inference. Gibbs sampling is a widely used inference technique and is a popular approach for parameter estimation and inference in many topic models such as LDA [7]. The advantage of using the Gibbs sampling method is that it is simple and easy to implement. In this study, Gibbs sampling is used to estimate the distributions of the latent variables. The pseudocode of the Gibbs sampling algorithm is given in Figure 6 for the proposed model, and the meanings of all variables are seen in Table 2. The algorithm will sample each variable (**z, s, q **, and **e**) based on the following formula by canceling terms in equations (2)–(6) (by replacing terms in (1) with those in equations (2)–(6):

(7) $$\begin{array}{c} P\left(z_{r,i}=z,s_{r,i}=s,q_{r,i}=q,e_{r,i}=e\;|z_{r,i},s_{r,i},q_{r,i},e_{r,i},\beta ,\alpha ,\gamma ,\mu ,\delta \right)\propto \frac{\left\{N_{w,z,s,q,e}\right\}+\beta }{\left\{N_{z,s,q,e}\right\}+\left|V\right|\times \beta }\times \frac{\left\{N_{z,s,r}\right\}+\alpha }{\left\{N_{s,r}\right\}+\left|Z\right|\times \alpha }\times \frac{\left\{F_{s,r}\right\}+\gamma }{\left\{F_{r}\right\}+\left|S\right|\times \gamma }\\ \times \frac{\left\{N_{q,z,r}\right\}+\mu }{\left\{N_{z,r}\right\}+\left|Q\right|\times \mu }\times \frac{\left\{N_{e,z,s,r}\right\}+\delta }{\left\{N_{z,s,r}\right\}+\left|E\right|\times \delta }, \end{array}$$

where **z**_*r*,*i*_, **s**_*r*,*i*_, **q**_*r*,*i*_, and **e**_*r*,*i*_ are topic, sentiment, window, and weight assignments, respectively, for all the words in the collection, except for the word considered at position *i* in document *r*. Posterior inference of parameters is performed using Gibbs sampling, as demonstrated in Figure 6.

In the section of initialization, the method randomly sets the parameters. A sentiment dictionary is employed for initializing sentiment labels. The sentiment dictionary contains words and scores that specify positive and negative labels and their weight. In this study, AFINN [64] is used as *a* sentiment dictionary, improving the model's accuracy. At the end of the sampling algorithm, each word has a weight and a sentiment label. Therefore, a dictionary can generate sentiment scores (weights and sentiment labels) and words. The scores are extracted from a dataset based on P(**w| s, e**). Each word's weight and sentiment with the most probability are selected as sentiment scores among all documents. Adaptive Lexicon learning using Genetic Algorithm (ALGA) [46] uses the genetic algorithm to generate a sentiment dictionary. However, we use topic modeling in WJST, to generate this dictionary. In WJST, the window size is different for various words. At each step of the sampling algorithm, count variables such as *F*_*s*,*r*_ and *F*_*r*_ are updated after sampling sentiment label, weight, and window size. After completing the sampling, the distribution of latent variables (*φ*, *θ*, *π*, *ξ*,  and *ψ*) can be calculated as follows:

(8) $$\begin{array}{c} \varphi =\frac{\left\{N_{w,z,s,q,e}\right\}+\beta }{\left\{N_{z,s,q,e}\right\}+\left|V\right|\times \beta }, \end{array}$$

(9) $$\begin{array}{c} \theta =\frac{\left\{N_{z,s,r}\right\}+\alpha }{\left\{N_{s,r}\right\}+\left|Z\right|\times \alpha }, \end{array}$$

(10) $$\begin{array}{c} \pi =\frac{\left\{F_{s,r}\right\}+\gamma }{\left\{F_{r}\right\}+\left|S\right|\times \gamma }, \end{array}$$

(11) $$\begin{array}{c} \xi =\frac{\left\{N_{q,z,r}\right\}+\mu }{\left\{N_{z,r}\right\}+\left|Q\right|\times \mu }, \end{array}$$

The probability of a word given a topic would be equal to$\sum_{s,q,e}P\left(w|z,s,q,e\right)$, and the probability of a sentiment label given a document for sentiment classification at the document-level is calculated using *π*.

The time complexity of the proposed method quantifies the amount of time taken by the Gibbs sampling algorithm to run as the main function. Given the number of words in all documents *w*_ALL_ (*w*_ALL_=∑_*r*∈*R*_*N*_*r*_, where *N*_*r*_ is the number of words in document *r*), the number of topics |*Z|*, the number of distinct windows |*Q|*, the number of weights |*E|*, and the total number of sentiment labels |*S|*, the time complexity of each Gibbs sampling iteration would be O(*w*_ALL_·*|S|·|Z|·|Q|·|E|*). Furthermore, given the number of iterations *G*, the total time complexity of WJST would be O(*G·w*_*ALL*_*·|S|·|Z|·|Q|·|E|*). Table 3 compares different methods in terms of time complexity.

### 3.4. The General Structure of WJST1

**Figure 7:**
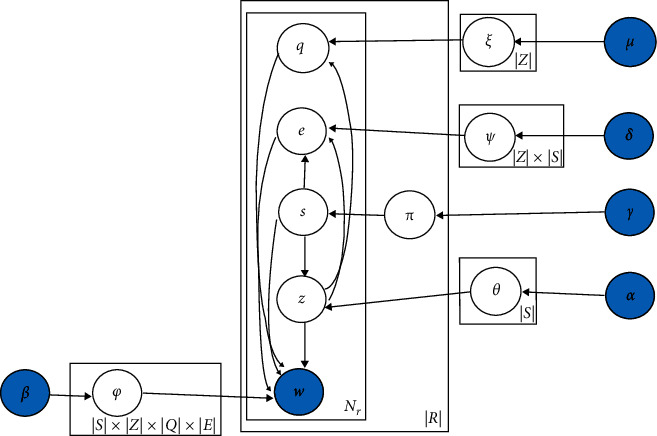
The graphical model of WJST1.

A version of WJST called WJST1 is presented in Figure 7. The distributions *θ*, *ξ*, and *ψ* in WJST depend on the document, but in WJST1, the distributions *θ*, *ξ*, and *ψ* do not rely on the document. Dependency between documents of a domain is more than documents in different domains. A pattern in documents of a domain may not exist in documents of other domains. So, calculations on multidomain datasets should be local and not cover all domains. For example, considering the distributions *P*(**z***| ***s**) and *P*(**z***| ***s**, **r**), where **z** is topic, **s** is sentiment, and **r** documents, in the first state *P*(**z***| ***s**), topic depends on sentiment. The distribution covers all documents in different domains. Perhaps a topic is positive in one domain and negative in another domain. So, it is better to depend the topic on the documents of a domain, not all domains. Thus, the topic is limited to the document (and domain), and contradiction between different domains is eliminated. So, WJST is suitable for multidomain datasets, and WJST1 is a version of WJST suitable for single-domain datasets. According to Figure 7, *ξ* is the probability of **q** given **z**, *θ* is the probability of **z** given **s**, and *ψ* is the probability of **e** given **z** and **s**, and the joint probability distribution for WJST1 can be factored as follows:

(12) $$\begin{array}{c} P\left(w,z,s,q,e\right)=P\left(w|z,s,q,e\right)\times P\left(z|s\right)\times P\left(s|r\right)\times P\left(q|z\right)\times P\left(e|z,s\right) \end{array}$$

where by integrating out *θ*, we achieve:

(13) $$\begin{array}{c} P\left(z|s\right)={\left(\frac{\Gamma \left(\left|Z\right|\times \alpha \right)}{\Gamma {\left(\alpha \right)}^{\left|Z\right|}}\right)}^{\left|S\right|}\prod_{s}\frac{\prod_{\text{z}}\Gamma \left(N_{z,s}+\alpha \right)}{\Gamma \left(N_{s}+\left(\left|Z\right|\times \alpha \right)\right)}, \end{array}$$

where *N*_*z*,*s*_ is the number of words with topic *z* and sentiment *s*. The symbol *N*_*s*_ is the number of words with sentiment *s*. The symbol *α* is Dirichlet before *θ*. And by integrating out *ξ*, we achieve:

(14) $$\begin{array}{c} P\left(q|z\right)={\left(\frac{\Gamma \left(\left|Q\right|\times \mu \right)}{\Gamma {\left(\mu \right)}^{\left|Q\right|}}\right)}^{\left|Z\right|}\prod_{z}\frac{\prod_{q}\Gamma \left(N_{q,z}+\mu \right)}{\Gamma \left(N_{z}+\left(\left|Q\right|\times \mu \right)\right)}, \end{array}$$

where *N*_*q*,*z*_ is the number of words with topic *z* and window *q*. The symbol *N*_*z*_ is the number of words with topic *z*. The symbol *μ*  is Dirichlet before *ξ*. And by integrating out *ψ*, we achieve:

(15) $$\begin{array}{c} P\left(e|z,s\right)={\left(\frac{\Gamma \left(\left|E\right|\times \delta \right)}{\Gamma {\left(\delta \right)}^{\left|E\right|}}\right)}^{\left|Z\right|\times \left|S\right|}\prod_{s}\prod_{z}\frac{\prod_{e}\Gamma \left(N_{e,z,s}+\delta \right)}{\Gamma \left(N_{z,s}+\left(E|\times \delta \right)\right)}, \end{array}$$

where *N*_*e*,*z*,*s*_ is the number of words with topic *z*, weight *e*, and sentiment *s*. The symbol *N*_*z*,*s*_ is the number of words with sentiment *s* and topic *z*. The symbol *δ* is Dirichlet before *ψ*. The symbols *P*(**w***| ***z**, **s**, **q**, **e**) and *P*(**s***| ***r**) are calculated using equations (2) and (4), respectively. After completing the sampling, the distribution of latent variables (*θ*, *ξ*, and *ψ*) is calculated as follows:

(16) $$\begin{array}{c} \theta =\frac{\left\{N_{z,s}\right\}+\alpha }{\left\{N_{s}\right\}+\left|Z\right|\times \alpha },\\ \xi =\frac{\left\{N_{q,z}\right\}+\mu }{\left\{N_{z}\right\}+\left|Q\right|\times \mu },\\ \psi =\frac{\left\{N_{e,z,s}\right\}+\delta }{\left\{N_{z,s}\right\}+\left|E\right|\times \delta }. \end{array}$$

And *φ* and *π* are computed through equations (8) and (10), respectively. Experimental results are demonstrated in the next section.

## 4. Experimental Results

The present study executes the methods on a computer with an Intel Core i7 CPU and 8 GB RAM. Proposed models are compared on 13 datasets. 4 datasets crawled from Amazon (https://www.amazon.com) opinions include Electronic, Movie, Android, and Automotive. 2 MDS datasets [65] contain Magazines and Sports. A dataset crawled from the IMDB movie archive [3] is MR. 3 UCI datasets [66] include Amazon, Yelp, and IMDB. 3 Twitter datasets [46] include STS-Test, SOMD, and Sanders. Data preprocessing contains (1) lowercasing all words, (2) removing digits, nonalphabetic characters, stop words, and words with too low and too high frequency, and (3) stemming. The details of the datasets are provided in Table 4.

The number of topics is unknown, provided as a constant amount at the beginning of the Gibbs sampling algorithm. In this study, *α*, *γ*,  *β*, and *δ* specific distributions are symmetric, and we empirically set the value of parameters, and this setting demonstrates fairly good performance in our experiments.  Table 5 exhibits the initialization of parameters used in different algorithms.

A sentiment dictionary is employed for initializing sentiment labels. Sentiment dictionaries such as AFINN [64], IMDB [67], 8-K [67], and Bing Liu [68, 69] contain words and scores that specify positive and negative labels as well as their weight. In the present study, AFINN is used as a sentiment dictionary which improves the model's accuracy. Sentiment detection at the document-level, perplexity, and topic_coherency are used to compare the efficacy of proposed models as three standard parameters which are used in different papers [7, 70, 71–73].

In the present study, the Accuracy parameter uses the formula of ((TP + TN))⁄((TP + FP + TN + FN)), where TP is the number of true positives, TN is the number of true negatives, FP is the number of false positives, and FN is the number of false negatives.

*π* distribution equation (10) determines how likely each comment is positive or negative. For example, if the value of *P*(+) is more significant than the value of *P*(−) (for a comment), the comment will be positive. The Accuracy's formula uses *π* distribution (equation (10)) to calculate TP, TN, FP, and FN values. For example, if a comment is positive and detected as positive (by the proposed methods), a unit is added to TP.

So, sentiment analysis (sentiment detection) at the document-level is realized using *π* distribution (equation (10)), and the formula of ((TP + TN))⁄((TP + FP + TN + FN)) is used to compute the Accuracy. 

The error formula can be calculated using the formula of (1-Accuracy). Accuracy, perplexity, and topic_coherency are used for evaluations in the present study. Further study can investigate more parameters such as MSE, MAE, and RMSE for future research.

Furthermore, Better methods have lower perplexity and also higher topic_coherency. Given a test dataset *D*_Test_, the perplexity is computed through

(17) $$\begin{array}{c} \text{Perplexity}\left(D_{\text{Test}}\right)=\text{exp}\left(\frac{-\sum_{r=1}^{\left|R\right|}\log\;P\left(w_{r}\right)}{\sum_{r=1}^{\left|R\right|}N_{r}}\right), \end{array}$$

where *w*_**r**_ are the words in document **r**, *N*_**r**_ is the length of document **r**, and *P*(*w*_**r**_) is the probability of words in document **r**. The lower value of the formula over a held-out document demonstrates Better generalization efficacy. The evaluation results are shown in Tables 6–8, 9–14, and the proposed models demonstrate better results. In the report of Tables 6–8, 9–14, the perplexity of proposed methods is lower than that of baseline models. In the report of Tables 9–12, the perplexity is reduced with an increase in topics. Topic_coherency is also calculated using

(18) $$\begin{array}{c} \text{Average Topic}-\text{Coherency}\left(Z\right)=\frac{\left(\sum_{i=1}^{\left|Z\right|}C\left(V^{\left(z_{i}\right)}\right)\right)}{\left|Z\right|}\\ =\frac{\left(\sum_{i=1}^{\left|Z\right|}\sum_{m=2}^{M}\sum_{n=1}^{m-1}\text{log}\left(\text{CODF}\left(v_{m}^{\left(z_{i}\right)},v_{n}^{\left(z_{i}\right)}\right)+1/\text{DF}\left(v_{n}^{\left(z_{i}\right)}\right)\right)\right)}{\left|Z\right|}. \end{array}$$

where *V*^(*z*_*i*_)^=(*v*_1_^(*z*_*i*_)^,…, *v*_*M*_^(*z*_*i*_)^) is the list of *M* words that have a high probability in the topic *z*_*i*_, *C*(*V*^(*z*_*i*_)^) is topic_coherency for the topic *z*_*i*_, *Z* is the set of all topics, |*Z*| is the number of distinct topics*, DF* is the document frequency, and *CODF* is the co-occurrence of two words in different documents. A smoothing count of 1 is included to avoid taking the logarithm of zero. In the present study, topic_coherency is computed through (18), equal to the average of topic_coherency values in *Z*. Furthermore, a higher value of topic_coherency reflects the better quality of the detected topics. *M* is equal to 10, and results are demonstrated in Tables 6–8, 9–14. A different number of topics (5, 10, 15, and 20) and different distinct windows (1, 2, 3, 4, 5, and 6) are applied for evaluating models. In this part, baseline methods include JST [8], RJST [10], TJST [8], and TS [9]. In the present section, the Friedman test [74, 75] is used to examine the achievements of the comparison methods. The Friedman test is a nonparametric multiple comparison test utilized to examine the differences between algorithms by assigning the lowest rank to the best approach in minimization problems and the highest rank to the best approach in maximization problems.

There are several methods for the validation of classification and topic modeling-based problems. Still, the methods used in this study are the most common and are used in most articles related to our article for evaluation. Also, there are various methods for validation that we will try to use in a future study to evaluate the proposed methods. The following is the reason for choosing the validation methods used in this study:

We chose accuracy, perplexity, and coherence score as evaluation metrics because of their popularity in classification and topic modeling problems. Perplexity is an essential metric that, in theory, represents how well a model behaved on unseen data and is provided using the normalized log-likelihood technique. Meanwhile, the coherence score measures the degree of semantic similarity between high-scoring words and helps distinguish the semantical interpretation of topics based on statistical inference.

The main question we want to answer is whether the proposed methods can improve the performance of text sentiment classification. This study compares proposed methods with different baselines, including JST and recently representative approaches. Consider a Confusion Matrix for a classification problem that predicts whether a comment has positive sentiment or not. The total number of correctly detected cases is one of the more obvious measures. When all of the classes are equally important, it is typically utilized. When True positives and True negatives are more significant, accuracy is employed. According to the accuracy criterion, one can immediately know whether the model is adequately trained or not and how it works in general. The most popular measurement for classification issues is accuracy, which is the proportion of correctly predicted cases to all cases. This metric's opposite, or error, can be calculated as 1-accuracy. In machine learning, an accuracy parameter is an excellent option for sentiment classification when the classes in the dataset are almost evenly distributed. Also, we will try to use various metrics such as recall and precision in future studies to evaluate the proposed methods.

We use the Friedman test to compare the results produced by the proposed methods and the competitors to verify the classification performance. Friedman's test is used to examine the achievements of the comparison methods. The Friedman test is a nonparametric multiple comparison test that is utilized to explore the differences between algorithms by assigning the lowest rank to the best approach in minimization problems and the highest rank to the best approach in maximization problems.

Topic modeling is one of the most important NLP fields. It aims to explain a textual dataset by decomposing it into two distributions: topics and words. A topic modeling algorithm is a mathematical or statistical model used to infer what the issues that better represent the data are. Human judgment-based review techniques can yield good results but are expensive and time-consuming. Human judgment is also not well defined. 

In contrast, the appeal of quantitative metrics such as perplexity is the ability to standardize, automate, and scale the evaluation of topic models. In natural language processing, perplexity is a traditional metric for evaluating topic models. The lower value of the formula over a held-out document demonstrates better generalization efficacy.

Perplexity's inability to capture context and the relationships between words within a topic or across topics within a document is one of its drawbacks. For human understanding, semantic context is important. Approaches like topic coherency have been designed to tackle this problem by capturing the context between words in a subject. Extracting topic words is one of the main tasks in topic modeling. In most articles about topic modeling, topic_coherency is shown as a number that represents the overall topics' interpretability and is used to assess the topics' quality. The higher the topic_coherency value, the better the quality of the subjects extracted.

### 4.1. Sentiment Scores for the Words in a Dataset

In this section, a dictionary is generated, including sentiment scores (weights and sentiment labels) and words. The scores are extracted from datasets based on P(**w| s, e**). The weight and sentiment with the most probability are selected for each word as a sentiment score. The extracted scores for some phrases in the form of unigram can be seen in Tables 15 and 16. ALGA [46] uses the genetic algorithm to generate a sentiment dictionary; however, we use topic modeling in the proposed models to create this dictionary. According to Tables 15 and 16, ten words from each dataset are selected and scored by the proposed models. For example, the word *nice* obtains a score of 4 in WJST and obtains a score of 5 in WJST1. The scores are different in the proposed methods; for example, the word *serious* achieves a score of 1 in WJST and a score of -2 in WJST1. Table 15 is related to Android, Automotive, Electronic, and Movie datasets.  Table 16 is associated with STS, Sanders, and SOMD datasets.

### 4.2. Topic Discovery

The topics are extracted from datasets based on *P*(*w|z*) in this section. A topic is a multinomial distribution over words based on topics, sentiments, weights, and window sizes. The top words could approximately reflect the meaning of a topic. Tables 17–19 show some examples of topics extracted from Movie, Android, and Electronic datasets by different models. Each row shows the top 10 words for the corresponding topic and sentiment label. The top 10 words from each topic were extracted and then used for topic_coherency. Extracting topic words is one of the main tasks in topic modeling. This section lists the top 10 words in three examples for Movie, Android, and Electronic datasets. The listed words for each topic describe the topic. The listed words for the proposed methods have a better topic_coherency value than baseline methods because they have a higher value of topic_coherency. The higher the topic_coherency value, the better the quality of the subjects extracted.

### 4.3. Sentiment Classification at Document-Level

**Figure 8:**
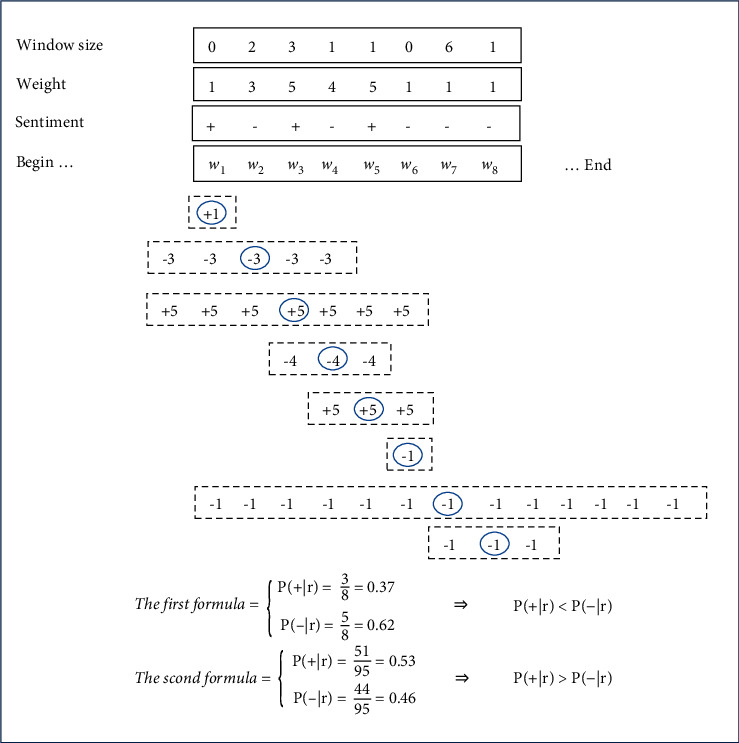
An example of calculating the sentiment of a document using two formulas.

In this section, the number of distinct windows is three, and the models use the AFINN sentiment dictionary in the initialization section of the Gibbs sampling algorithm. A document is classified based on *P*(**s**| **r**), which is the probability of a sentiment given by a document. A document is classified as negative if P(+|**r**) < P(−| **r**) and vice versa. Determining sentiment is important which is calculated using two formulas in this paper. In the first formula, P(**s***| ***r**)=*N*_*s*,*r*_/*N*_*r*_ where *N*_*s*,*r*_ is the number of words with sentiment *s* in document *r* and *N*_*r*_ is the number of words in document *r*. In the second formula, P(**s***| ***r**)=*F*_*s*,*r*_/*F*_*r*_ where *F*_*s*,*r*_ is the effect of words with sentiment *s* in document *r* and *F*_*r*_ is equal to the sum of the effect of words with different sentiments in document *r*. In all evaluations, accuracy1 is calculated based on the first formula, and accuracy2 is calculated based on the second formula. As shown in Figure 8, the document is negative according to the first formula, and the document is positive according to the second formula, and the weight of positive words is more than negative ones, although the number of negative words is more than positive ones, and positive words can affect sentiment analysis at document-level.

In this section, the best values for each method (the highest accuracy, the lowest perplexity, and the highest topic_coherency) are selected from Tables 9–12 and are listed in Tables 6 and 7.  Table 6 compares the models based on four datasets (Android, Automotive, Movie, and Electronic) and Table 7 compares the models based on six datasets (Magazine, Sports, MR, Amazon, IMDB, and Yelp). The results of Tables 6 and 7 are evaluated on unigram words. AFINN method classifies each document according to the P(s*|*r)=*N*_*s*,*r*_/*N*_*r*_ where the word sentiment label is directly obtained from the AFINN sentiment lexicon. The RND method classifies each document according to the P(s*|*r)=*N*_*s*,*r*_/*N*_*r*_ where the word sentiment label is determined randomly, and in the AFINN + RND method, the algorithm uses both AFINN and RND methods. The improvement over these methods will reﬂect how much the proposed methods and baseline methods can learn from a dataset. The report in Tables 6 and 7 shows that the proposed models perform better than JST. Based on the results, the proposed methods have a signiﬁcant improvement over AFINN and the baseline methods on all datasets. As seen from AFINN-based methods results, the results calculated based on the sentiment lexicon are below 70% for most datasets. In this study, parameters perplexity and topic_coherency are not calculated for AFINN, RND, and AFINN + RND methods. TS and RJST methods have lower accuracy than other methods on all datasets, but JST and TJST achieve better performance. As can be seen from the results, TJST outperforms JST on all datasets because, in JST, the distribution *θ* depends on the document, but in TJST, the distribution *θ* does not depend on the document and is generally estimated because it uses all documents for computations. According to Tables 6 and 7, WJST1 has higher accuracy than other methods. WJST1 outperforms WJST because, in WJST, the distributions *θ*, *ξ*, and *ψ* depend on the document, but in WJST1, the distributions *θ*, *ξ*, and *ψ* do not depend on the document and are generally estimated because they use all documents for computations. The perplexity value varies on different datasets because the size of datasets is different, according to Table 4.

**Figure 9:**
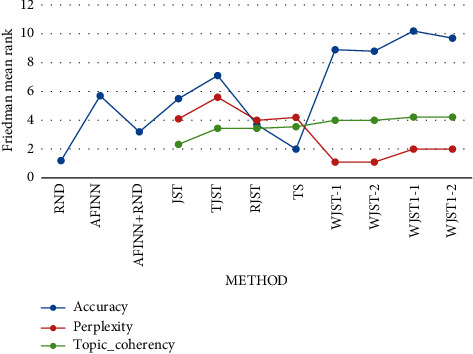
According to the Friedman test, the mean rank of algorithms in Tables 6 and 7.

**Figure 10:**
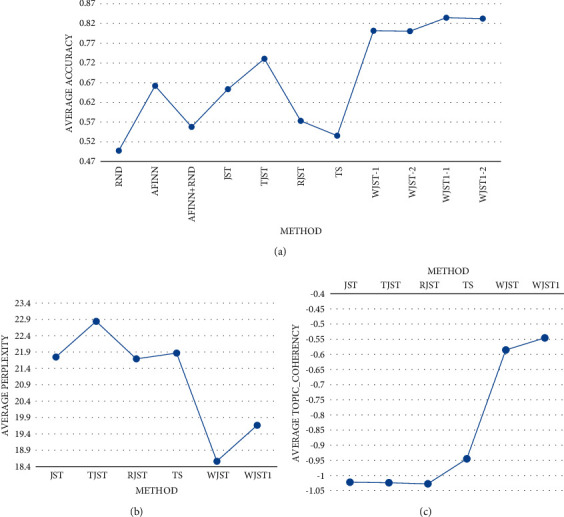
Average of sentiment classification values calculated on Android, Automotive, Electronic, Movie, Magazine, Sport, MR, Amazon, IMDB, and Yelp datasets (based on Tables 6 and 7), in terms of accuracy (a), perplexity (b), and topic_coherency (c).

The analysis of the Friedman test on the results of Tables 6 and 7 demonstrates that there is a statistically significant difference between the performances of the algorithms in terms of accuracy with *χ*^2^(10)=92.091 and *p* < 0.01, in terms of perplexity with *χ*^2^(5)=38.629 and *p* < 0.01, and in terms of topic_coherency with *χ*^2^(5)=5.508 and *p* > 0.1. The mean rank of the algorithms based on the Friedman test, which is demonstrated in Figure 9, indicates that WJST1 ranks first among all the algorithms in 7 in terms of accuracy and topic_coherency. According to Figure 9, if the experiment intends to find the minimum value (perplexity), the Friedman test assigns the lowest rank to the best-performing algorithm. If the problem intends to find the maximum value (accuracy and topic_coherency), the Friedman test assigns the highest rank to the best-performing algorithm. According to Figure 9, *-1* is accuracry1 and *-2* is accuracy2. As shown in Figure 10, average values of accuracy, perplexity, and topic_coherency are equal to the average values in each column of Tables 6 and 7 for each method, in which the values are calculated on Android, Automotive, Electronic, Movie, Magazine, Sport, MR, Amazon, IMDB, and Yelp datasets. According to the results, WJST has a lower perplexity value than other methods. WJST1 outperforms WJST and baseline methods in terms of accuracy and topic_coherency. According to Figure 10, *-1* is accuracry1 and *-2* is accuracy2.

### 4.4. Evaluation Results According to the Different Situations, with AFINN and NO_AFINN States

In this section, the study aims to examine the impact of the AFINN dictionary in the initialization part of Gibbs sampling on the proposed models. The results of the evaluation are shown in Table 8. In this section, the number of distinct windows is three, and the number of topics is ten. The most effective is visible in WJST1 on the Movie dataset, where the accuracy in the NO_AFINN state is equal to 0.48 and is equal to 0.95 in the AFINN state. Prior sentiment information affects perplexity and topic_coherency lower than accuracy. According to Table 8, it can be seen that using the AFINN dictionary is more effective than using the NO_AFINN state. In the NO_AFINN state, prior sentiment information was not incorporated into the models for sentiment words in the initialization section of the Gibbs sampling algorithm.

### 4.5. Evaluation Results According to the Different Sentiment Dictionaries

In this subsection, the study compares different dictionaries achieved by the proposed models. The output of WJST and WJST1 can be a weighted sentiment dictionary. Using the obtained dictionary by each method on each dataset, other datasets will be evaluated. Each document will be classified according to *P*(**s|r**), where the word sentiment label is directly obtained from the dictionary. Tables 20 and 21 are related to WJST and WJST1, respectively. The impact of using different dictionaries achieved by WJST and WJST1 is presented in Tables 20 and 21. The methods AFINN + *w*, Android + *w*, ELEC + *w*, Auto + *w*, and MOV + *w* classify each document according to P(s*|*r) = *F*_*s*,*r*_/*F*_*r*_, where the weight and sentiment label is directly obtained from AFINN, Android, Electronic, Automotive, and Movie lexicons, and window size is considered to be one for all words in all documents. Methods Bing_Liu, 8K, Android, Automotive, ELEC, MOV, and IMDB classify each document according to P(s*|*r) = *N*_*s*,*r*_/*N*_*r*_ where the word sentiment label is directly obtained from Bing_Liu, 8K, Android, Automotive, Electronic, Movie, and IMDB lexicons, respectively. The Bing_Liu + RND method uses both Bing_Liu and RND methods. In the IMDB + RND method, the algorithm uses both IMDB and RND methods. The 8K + RND method utilizes both 8K and RND methods. In the Android + RND method, the algorithm uses both Android and RND methods. In the Auto + RND method, the algorithm uses both Auto and RND methods. In the ELEC + RND method, the algorithm uses both ELEC and RND methods. In the MOV + RND method, the algorithm uses both MOV and RND methods. According to Table 20, the AFINN method achieves the highest accuracy on one dataset. Proposed methods achieve the highest accuracy on six datasets. According to the results in Table 21, the proposed methods achieve the highest accuracy on seven datasets. In AFINN, Bing Liu, IMDB, and 8-k dictionaries, sentiment and score values are set manually for each word, but proposed models use topic modeling to generate dictionaries. Proposed methods such as MOV and ELEC perform well on the datasets on which they are created based on. The dictionaries achieved by the proposed models are dependent on the application domain.

**Figure 11:**
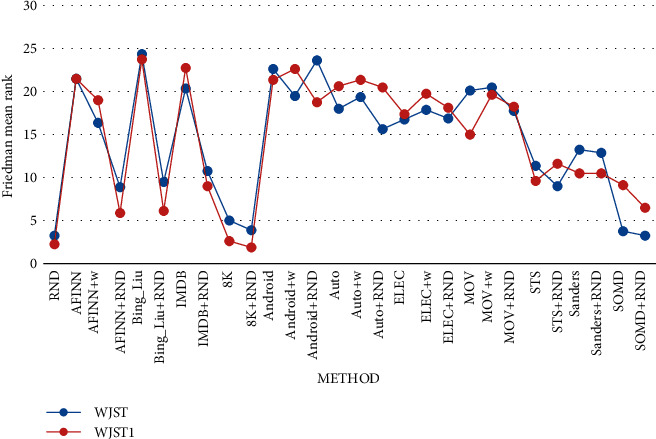
The mean rank according to the Friedman test based on results of Tables 20 and 21.

**Figure 12:**
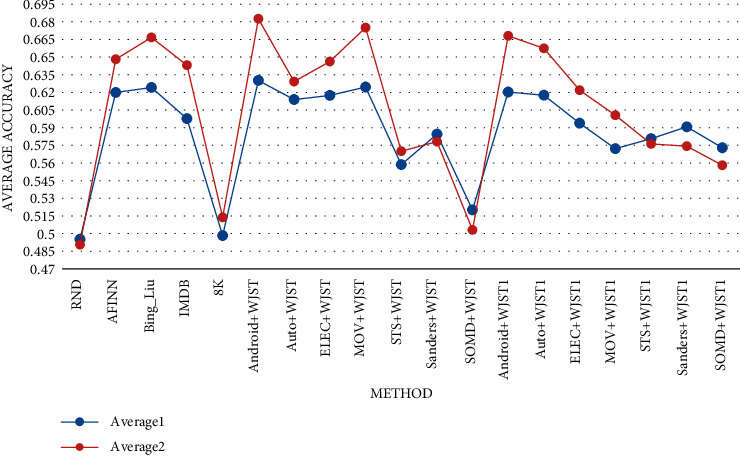
Average accuracy values are calculated using different sentiment dictionaries on Android, Automotive, Electronic, Movie, STS, Sanders, and SOMD datasets (based on Tables 20 and 21).

The analysis of the Friedman test on the results of Table 20 demonstrates that there is a statistically significant difference between the performances of competitors in terms of accuracy with *χ*^2^(27)=70.070 and *p* < 0.01. The analysis also shows that there is a statistically significant difference between the performances of the algorithms in Table 21 in terms of accuracy with *χ*^2^(27)=79.740 and *p* < 0.01. The mean rank of the algorithms can be seen in Figure 11. As shown in Figure 12, *Average1* is equal to the average of values in each row for each method, in which the values are calculated on datasets Android. Automotive, Electronic, Movie, STS, Sanders, and SOMD based on Tables 20 and 21. Furthermore, *Average2* is equal to the average of values in each row for each method, in which the values are calculated on Android, Automotive, Electronic, and Movie based on Tables 20 and 21. According to the results, Bing_Liu achieves the highest value on column *Average1*. Furthermore, the Android, MOV, and Bing_Liu methods have higher accuracy than other methods.

### 4.6. Evaluation Results According to the Different Number of Topics

In this subsection, the proposed models are examined based on the different topics (5, 10, 15, and 20). The AFINN dictionary is utilized in methods, and the number of distinct windows is three. Evaluations results are shown in Tables 9–12. The proposed methods are better than the baseline methods based on the results. The results show that increasing the number of topics will decrease the perplexity value. WJST1 achieves the highest accuracy on the Movie dataset with 97 percent, but the highest accuracy value on the Movie dataset in WJST is equal to 84 percent. Results show that the proposed methods perform better with different topic number settings, especially for WJST1 with 97% accuracy at |*Z*| = 5 on the Movie dataset. Based on the results, WJST has a lower perplexity than other methods. WJST1 outperforms WJST and baseline methods in terms of accuracy and topic_coherency. TJST performs better than the WJST method in terms of accuracy, but WJST achieves higher accuracy than JST and other baseline methods. This observation shows that modeling the parameters *weight* and *window* improves sentiment classification at the document-level. According to (18), a lower topic_coherency value suggests that the retrieved subjects are of worse quality than one with a highertopic_coherency. The words in a subject accurately describe the subject and have a stronger association with one another. 

### 4.7. Evaluations Results According to the Different Number of Distinct Windows

In this subsection, the proposed models are evaluated according to the different number of separate windows (1, 2, 3, 4, 5, and 6), which are effective for improving the proposed models. In this experiment, the number of topics is five, and the models use the AFINN sentiment dictionary. Based on Table 13, the proposed methods are compared according to accuracy, perplexity, and topic_coherency. The results show that increasing the number of distinct windows will decrease the perplexity value. In the report in Table 13, an increase in the size of a window will reduce the accuracy because it will increase the number of words in the window, and each term may not affect all neighbors in its window.

**Figure 13:**
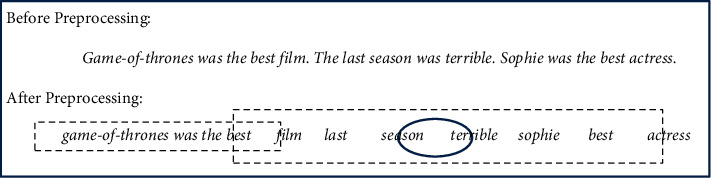
An example for showing the effect of each word on neighbors in its window.

For instance, as shown in Figure 13, the word *terrible* has a window size equal to 3. In Table 13, it is assumed that each word affects all neighbors in its window, so Table 13 takes that the unigram *terrible* effects unigrams *film, last, season, sophie, best,* and *actress.* As shown in Figure 13, the word *terrible* can affect unigrams *film, last,* and *season,* but it is not about unigrams *sophie, best,* and *actress.* So, finding the words that can be involved in a window is a new challenge that we introduce in this study, and two methods are presented. The first method assumes that each word affects all neighbors in its window, and the second method assumes that each word affects some random neighbors in its window. So, the first method selects all neighbors, but the second method selects some neighbors randomly. In this study, all evaluations are calculated based on the first method, and the second method is considered for the evaluation in Table 14.

As shown in Table 13, accuracy, perplexity, and topic_coherency values are calculated on Android, Automotive, Electronic, and Movie datasets before random selection using the first method. As shown in Table 14, accuracy, perplexity, and topic_coherency values are calculated on Android, Automotive, Electronic, and Movie datasets after random selection using the second method. So, in Table 13, it is assumed that each word affects all neighbors in its window, but in Table 14, it is assumed that each word affects some random neighbors in its window. As shown in Tables 13 and 14, average values of accuracy, perplexity, and topic_coherency are equal to average values in each column of Tables 13 and 14 for each window size. The values are calculated on Android Automotive, Electronic, and Movie datasets. According to the results, the second method is more stable than the first method in terms of accuracy, but the first method has higher accuracy than the second method. The second method outperforms the first method in terms of perplexity. The first method performs better than the second in terms of accuracy and topic_coherency.

**Figure 14:**
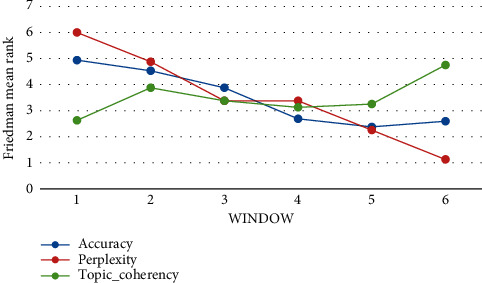
The mean rank of the seven algorithms is in Table 13 according to the Friedman test.

**Figure 15:**
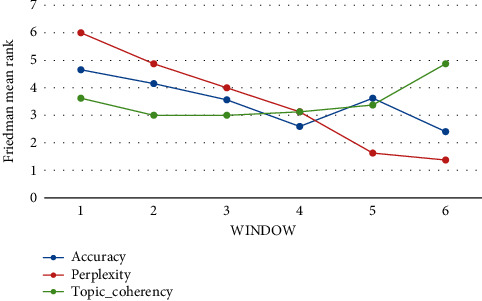
The mean rank of algorithms is in Table 14 according to the Friedman test.

The analysis of the Friedman test on the results of Table 13 demonstrates that there is a statistically significant difference between the performances of the algorithms in terms of accuracy with *χ*^2^(5)=27.608 and *p* < 0.01, in terms of perplexity with *χ*^2^(5)=35.143 and *p* < 0.01, and in terms of topic_coherency with *χ*^2^(5)=6.232 and *p*=0.284. The mean rank of the algorithms based on the Friedman test, which is demonstrated in Figure 14, indicates that (*w* = 1) outperforms other windows in terms of accuracy. Still, it has lower perplexity and topic_coherency values than other windows. (*w* = 6) outperforms other windows in perplexity and topic_coherency, but it has a lower accuracy value than other windows. (*w* = 3) provides a special situation for proposed algorithms in which accuracy, topic_coherency, and perplexity values are between the highest and lowest values (*w* = 1, 2, 4, 5, and 6). So, in this study, all evaluations are calculated based on (*w* = 3). The mean rank of the algorithms based on Table 14, which is demonstrated in Figure 15, indicates that (*w* = 1) ranks first among all the algorithms in Table 14 in terms of accuracy. The analysis of the Friedman test indicates that there is a statistically significant difference in terms of accuracy with *χ*^2^(5)=17.550 and *p* < 0.01, in terms of perplexity with *χ*^2^(5)=37.857 and *p* < 0.01, and in terms of topic_coherency with *χ*^2^(5)=5.857 and *p*=0.320.

### 4.8. Sentiment Classification Using Proposed Methods in Comparison to ALGA

**Figure 16:**
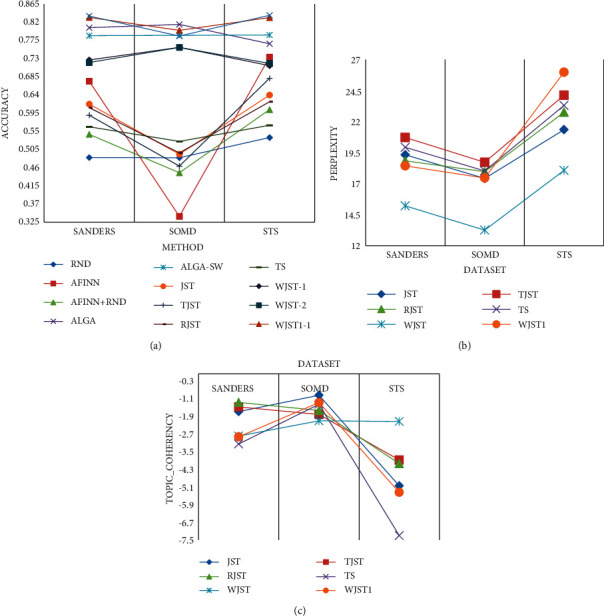
Sentiment classification on SANDERS, SOMD, and STS datasets, in terms of accuracy (a), perplexity (b), and topic_coherency (c).

In this subsection, WJST and WJST1 are compared to ALGA [46]. Three datasets have been selected for evaluating the methods. Evaluations results are shown in Figure 16, which compares the results of models with each other according to accuracy, perplexity, and topic_coherency metrics. In ALGA [46], several sentiment lexicons are created for a dataset during the training stage using a genetic algorithm. During the testing process, these dictionaries are employed. Every dictionary has some words and scores. Each chromosome is represented as a vector of sentiment words and their scores in the genetic algorithm employed in the method. The scores are spread between a feeling word's lowest and maximum scores. The primary goal of ALGA is to create a dictionary that reduces errors on training datasets. The sum of scores for words of each instance *T*_*i*_ in dataset *D*_*m*_ using dictionary *L*_*k*_ is calculated using equation (19) and is treated as a feature [46]:

(19) $$\begin{array}{c} \text{ALGA}\left(D_{m},T_{i},L_{k}\right)=\sum_{W_{j}\in T_{i}}v_{k}\left(W_{j}\right). \end{array}$$

Finding the values of words in the dictionaries (chromosomes) and adding them together is how the ALGA value for each instance is calculated. In (19), *W*_*j*_ represents the words of *T*_*i*_, and *v*_*k*_(*W*_*j*_) shows the score of *W*_*j*_ in *L*_*k*_. As mentioned in [46], ALGA will predict a positive instance when the ALGA feature is positive and a negative instance when the ALGA feature is negative. By dividing the number of correct predictions of instances of a given dataset by the total cases, ALGA's accuracy is calculated. In this subsection, proposed methods are compared with ALGA [46] because it can automatically generate a sentiment dictionary. ALGA generates a sentiment dictionary using the genetic algorithm, but proposed methods generate a sentiment dictionary using topic modeling. In proposed models, each document is classified based on *P*(**s| r**), the probability of sentiment label given a document. In proposed models, two labels (+, −) are considered, and a document is classified as negative if P(+|**r**) < *P*(−|**r**) and vice versa. Evaluations results can be seen in Figure 16. In this subsection, the number of distinct windows is three, and the number of topics is five. The models use the AFINN sentiment dictionary. According to Figures 16(a)–16(c), each column compares different methods on a dataset. The details of the datasets used in this section are illustrated in Table 4. In this subsection, only the accuracy is considered for the evaluation of ALGA. The ALGA-SW value is achieved by executing ALGA without taking stopwords into account. According to the results, WJST has higher accuracy than TJST on all datasets. WJST1 outperforms WJST and TJST on all datasets. ALGA and ALGA-SW perform better than other methods in terms of accuracy, but WJST1 achieves higher accuracy than ALGA and ALGA-SW on STS and Sanders datasets. The RND method achieves the lowest accuracy value on Sanders and STS datasets. The AFINN + RND method has higher accuracy than the RND method and has a lower accuracy than the AFINN method on Sanders and STS datasets. The RND method outperforms TJST, AFINN, and AFINN + RND methods on the SOMD dataset. In this study, parameters perplexity and topic_coherency are not calculated for ALGA, ALGA-SW, AFINN, RND, and AFINN + RND methods. According to Figure 16, *-1* is accuracry1, and *-2* is accuracy2.

### 4.9. Sentiment Classification Using Proposed Methods on Multidomain Datasets

In this subsection, the performance of the proposed methods is compared with baseline methods on a multidomain dataset. In this experiment, the number of distinct windows and topics is three and five, respectively, and the models use the AFINN sentiment dictionary. The multidomain dataset contains reviews taken from multiple domains (product types). The details of the multidomain dataset used in this section are illustrated in Table 22.

**Figure 17:**
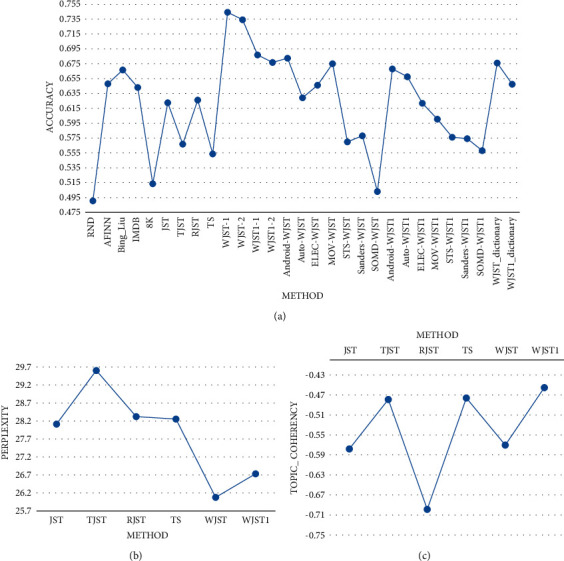
Sentiment classification values are calculated on a multidomain dataset in terms of accuracy (a), perplexity (b), and topic_coherency (c).

As shown in Figure 17, accuracy, perplexity, and topic_coherency values are calculated on a multidomain dataset that contains Android. Automotive, Electronic, and Movie domains. The methods WJST-dictionary and WJST1-dictionary classify each document according to P(s*|*r)=*N*_*s*,*r*_/*N*_*r*_. The word sentiment label is directly obtained from WJST and WJST1 lexicons achieved by WJST and WJST1 on the multidomain dataset. According to Figure 17, *-1* is accuracry1 and *-2* is accuracy2. Based on the results, WJST has a lower perplexity value than other methods. WJST1 outperforms WJST in terms of topic_coherency. WJST performs better than the WJST1 method in terms of accuracy because the distributions *θ*, *ξ*,  and *ψ* in WJST depend on document, but in WJST1, the distributions *θ*, *ξ*,  and *ψ* do not depend on the document. Dependency between documents of a domain is more than documents in different domains. A pattern in the documents of a domain may not exist in the documents of other domains. Therefore, calculations on multidomain datasets should be local and not cover all domains. For example, considering the distributions *P*(**z***| ***s**) and *P*(**z***| ***s**, **r**), where **z** is topic, **s** is the sentiment, and **r** is documents. In the first state (*P*(**z***| ***s**)), the topic depends on sentiment, and the distribution covers all documents in different domains. Perhaps a topic was positive in one domain and negative in another. Therefore, it is better to depend the topic on the documents of a domain, not all domains. Thus, the topic is limited to the document (and domain), and contradiction between different domains is eliminated. Therefore, WJST is suitable for multidomain datasets, and WJST1 is a version of WJST suitable for single-domain datasets. Sentiment classification on multidomain datasets is a challenge, and our solution in this study is using WJST, whose distributions (*θ*, *ξ*, and *ψ*) depend on the document. Sentiment classification on multidomain datasets is a challenge, and further studies can be conducted to investigate this problem for future research.

### 4.10. Comparison with Other Methods

In this subsection, the best performance of the proposed methods is compared with 57 competitors [13, 76]; [82–88], [8–10, 46] which is shown in Table 23. The details of the datasets used in this section are illustrated in Table 4.

### 4.11. Comparison with Discriminative Models

The proposed methods are compared to baseline approaches such as logistic regression and SVM on four datasets in the following experiment. The multidomain dataset contains Android, Automotive, Electronic, and Movie domains. As shown in Table 24, the accuracy value is calculated on four datasets. The results demonstrate that proposed methods have improved notably over AFINN and the baseline methods on all datasets. Based on Table 24, methods use two systems in preprocessing phase, which includes Bag of Word (BOW) and Term Frequency Inverse Document Frequency (TF-IDF). In the BOW system, more word frequency reflects more importance of the word. TF-IDF system believes that high frequency may not be able to provide much information. Furthermore, rare words contribute more weight to the method. According to evaluation results, the results of the TF-IDF system are better than the BOW system.

### 4.12. Comparison with JST According to Extended Features

Suppose a unigram corresponds to the sentiment lexicon. In that case, its polarity will be equal to the subjectivity of the lexicon in order to identify the emotion label of the unigram for trying to prepare prior emotion information. The following technique is used to decide the emotion label of a bigram to prepare prior emotion information: If words of the bigram have the same polarity, the bigram's polarity will be the same as that of the words. If one of the words is in the lexicon, the bigram's polarity will equal the lexicon's subjectivity. The bigram's polarity will be opposed to the polarity of the second word if the first word is ‘not.' The following methodology is used to decide the emotion label of a trigram in order to prepare prior emotion information. If words of the trigram have the same polarity, the trigram's polarity will be the same as that of the words. If one of them is in the lexicon, the trigram's polarity will equal the lexicon's subjectivity. The trigram's polarity will be opposed to the second or third word's polarity if the first or second word is ‘not.' The proposed methods are compared with JST on four datasets (single-domain) according to extended features (bigrams and trigrams) in the following experiment.

As shown in Table 25, the accuracy value is calculated on four datasets, and the experiment extends the features to bigrams and trigrams. According to the results, WJST1 outperforms WJST. According to evaluations results, proposed models outperform JST because the additional parameters can influence the process of producing words in a review appropriately. The perplexity value varies on different datasets because the size of datasets is different. As the number of grams increases, perplexity is increased because in each document, in addition to the unigrams (+bigrams), bigrams (+trigrams) are added to the data, and the size of the dataset is increased. As the number of grams increases, accuracy is improved. In some cases, it gets worse because higher grams (bigram or trigrams) are sometimes meaningless.

### 4.13. Discussions on the Limitations of the Proposed Methods

Although the analysis of the results of the evaluation can demonstrate the best performance of proposed methods, proposed methods have some limitations, as follows:

1. The first limitation is the time complexity of the proposed methods (O(*G·w*_ALL_*·|S|·|Z|·|Q|·|E|*)) which is more than baseline methods (O(*G·w*_ALL_*·|S|·|Z|*)) according to Table 3 in Section 3.3.
2. The second limitation is the window size. On the report in Table 13, an increase in the size of a window will decrease the accuracy because it will increase the number of words in the window, and each term may not affect all neighbors in its window. Therefore, finding the words that can be involved in a window is a new challenge that we introduce in this study, and two methods are presented in Section 4.7. The first method assumes that each word affects all neighbors in its window, and the second method assumes that each word affects some random neighbors in its window.Therefore, the first method selects all neighbors, but the second method selects some neighbors randomly. According to the results, the second method is more stable than the first method in terms of accuracy, but the first method has a higher accuracy value than the second method. The second method outperforms the first method in terms of perplexity. The first method performs better than the second in terms of accuracy and topic_coherency.

### 4.14. A Concise Description of the Proposed Solutions and the Results

The main problem in this study is to examine a user's opinion about a product or movie, for example. This means identifying whether a user has a positive or negative idea about a subject (a product or movie).

Two novel models have been proposed that use topic modeling to solve the above problem. So, our solution is using a technique named topic modeling. Proposed models extend and improve JST (as a topic model) through two new parameters. To improve JST, proposed models consider the effect of words on each other. The new parameters have an immense effect on model accuracy regarding evaluation results. According to evaluations results, the proposed models outperform JST because the additional parameters can influence the process of producing words in a review appropriately. They can improve sentiment classiﬁcation at the document-level. Also, in the evaluation results report, proposed methods are more accurate than discriminative models such as SVM and logistic regression. Proposed methods are more flexible than discriminative models because other information, such as the top 10 words, can be extracted from the heart of the data.

## 5. Conclusion

In this study, two new models called WJST and WJST1 have been presented that extend JST and improve accuracy metrics. Reviewing the various articles about sentiment analysis indicates that the proposed models are associated with innovation and lead to remarkable results compared to the baseline methods. The proposed models can generate a sentiment dictionary. According to evaluations results, the proposed models consider the effect of words on each other using the extra parameters, which are important and influential. The evaluation results indicate that the accuracy has been improved compared to the baseline methods such as JST, RJST, TS, TJST, and ALGA. Results show that the proposed methods perform better with different topic number settings. WJST1 outperforms other methods in terms of accuracy, demonstrating its effectiveness of that. Prior sentiment information affects perplexity and topic_coherency lower than accuracy.

According to evaluations results, using the AFINN dictionary as prior sentiment information is more effective than using the NO_AFINN state. ALGA uses the genetic algorithm to generate a sentiment dictionary; however, proposed methods use topic modeling to generate this dictionary. According to the evaluation results, the proposed models outperform JST because the additional parameters could influence the process of producing words in a review appropriately. They have the potential to increase the emotion detection's accuracy at the level of the document. The proposed methods are unsupervised, and no labeled data is required. Proposed methods can automatically assess web comments and categorize reviews as positive or negative. The proposed methods have tried to increase the accuracy with fewer parameters and, at the same time, simplicity compared to the existing methods. The proposed methods both analyze emotions at the document-level and create an emotional dictionary. They are also the first methods to create an emotional dictionary through a topic modeling technique and in an automatic and accurate way. The proposed methods are the first methods that consider the words in the text and their effect on each other in a dynamic and weighty way. Also, they are parametric.

## 6. Future Work

The proposed models are parametric in the present study, and further studies will be conducted to investigate nonparametric models. Sentiment classification on multidomain datasets is a challenge, and further studies can be conducted to investigate this problem for future research. In future research, the proposed methods can be evaluated on more datasets. More parameters can also be assessed. Twitter social network data have obtained significant attention in natural language processing studies, with certain conditions, such as short data length. In future articles, the proposed methods can be modified to analyze emotions with specific Twitter data.

## Figures and Tables

**Figure 6:**
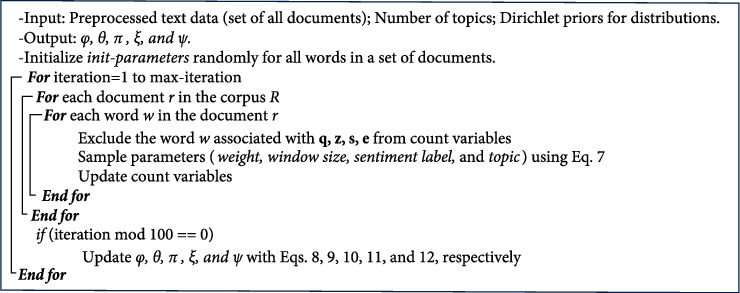
Adopted Gibbs sampling for WJST1.

**Table 1:** A general comparison of similar methods in recent years.

References	Method	Language	Dataset	General result
Pathak et al. [47]	Deep learning + topic modeling	English	Facebook, Ethereum, Bitcoin, SemEval-2017	Facebook-0.79, Ethereum-0.844, Bitcoin-0.817, SemEval-2017-0.889
Tang et al.[62]	Topic modeling	English	Amazon, Yelp	Amazon-0.82, Yelp-0.84
Kalarani and Selva Brunda [49]	Joint sentiment-topic features + POS tagging + SVM and ANN	English	Balanced dataset, unbalanced data	SVM-0.84, ANN-0.87
Farkhod et al. [50]	Topic modeling	English	IMDB	IMDB-F1 score-70.0
Fatemi and Safayani [51]	Topic modeling + restricted Boltzmann machine	English	20-Newsgroups (20NG), movie review (MR), multidomain sentiment (MDS)	Perplexity: MR: 406.74
Pathik and Shukla [53]	Deep learning + topic modeling	English	Yelp, Amazon, IMDB	Yelp- 0.75, Amazon-0.76, IMDB- 0.82
Sengupta et al.[54]	Topic modeling	English	Movies, Twitter	Perplexity: Movies- 3834.7, Twitter- 280.75
Huang et al.[56]	Deep learning	English	IMDB, Yelp	IMDB-0.963, Yelp-0.735
Özyurt and Akcayol [57]	Topic modeling	English + Turkish	User reviews in Turkish language about smartphones, SemEval-2016, Task-5 Turkish restaurant reviews	Precision-81.36 Recall-83.43 *F*-score-82.39
Zhao et al. [58]	Deep learning	English	Amazon review	CNN-87.7, LSTM-87.9
Rao et al. [59]	Deep learning	English	Yelp 2014, 2015, IMDb	Yelp2014–63.9 Yelp2015–63.8, IMDb-44.3
Naseem et al. [60]	Deep learning	English	Airline dataset	Airline dataset = 0.95
Basiri et al. [61]	Attention-based deep learning	English	Sentiment140, Airline, Kindle dataset, movie review	Kindle dataset = 0.93, Airline = 0.92, movie review = 0.90, Sentiment140 = 0.81

The proposed models are deeply described step-by-step in the next section.

**Table 2:** A summary of notations used in WJST.

*Symbol*	Description
*Collections*	
*R*	Set of all documents
*V*	Vocabulary set
*Q*	Set of all distinct windows (with different sizes)
*Z*	Set of all topics
*E*	Set of all distinct weights
*S*	Set of all sentiment labels

*Init parameters*	
**q**	Window variable
**e**	Weight variable
**r**	Document variable
**z**	Topic variable
**w**	Word variable
**s**	Sentiment variable

*Distributions*	
*θ*	Probability of **z** given **s** and **r**
*φ*	Probability of **w** given **z**, **s**, **q**, and **e**
*π*	Probability of **s** given **r** (**s**_0_ = Positive label, **s**_1_ = Negative label)
*ψ*	Probability of **e** given **z**, **s**, and **r**
*ξ*	Probability of **q** given **z** and **r**

*Hyper parameters*	
*α*	Dirichlet prior distribution for *θ*
*β*	Dirichlet prior distribution for *φ*
*γ*	Dirichlet prior distribution for *π*
*δ*	Dirichlet prior distribution for *ψ*
*μ*	Dirichlet prior distribution for *ξ*

**Table 3:** The time complexity of different models.

Model	Time complexity
JST, RJST, TJST, and TS	O(G· *w*_ALL_·|S|·|Z|)
WJST	O(G· *w*_ALL_·|S|·|Z|·|Q|·|E|)

**Table 4:** Description of datasets.

#	Dataset	Number of reviews	Vocabulary size	Number of words
1	Movie	400	6592	41540
2	Electronic	400	4501	29117
3	Automotive	400	3590	19733
4	Android	400	2173	9723
5	STS	359	1489	3784
6	SOMD	916	2013	7772
7	Sanders	1224	3221	14100
8	Magazines	1800	8040	125387
9	Sports	2000	8582	113921
10	MR	2000	33054	733022
11	Amazon	1000	1521	7296
12	IMDB	1000	2556	9706
13	Yelp	1000	1679	7726

**Table 5:** Initial values of parameters.

Model	Parameters
JST	Max_iteration:5000; |Z| = 5,10,15,20; *α*=0.1; *γ*=0.016 × (average document length); *β*=0.01;
RJST	Max_iteration:5000; |*Z*| = 5,10,15,20; *α*=0.1; *γ*=0.016 × (average document length); *β*=0.01;
TJST	Max_iteration:5000; |*Z*| = 5,10,15,20; *α*=0.1; *γ*=0.016 × (average document length); *β*=0.01;
TS	Max_iteration:5000; |*Z*| = 5,10,15,20; *α*=0.1; *γ*=0.016 × (average document length); *β*=0.01;
WJST	Max_iteration:5000; |*Z*| = 5,10,15,20; *α*=0.3; *γ*=0.016 × (average document length); *β*=0.01; *μ*=3; *δ*=9; *E* = [−5, +5]; *Q* = {1,2,3,4,5,6};
WJST1	Max_iteration:5000; |*Z*| = 5,10,15,20; *α*=0.3; *γ*=0.016 × (average document length); *β*=0.01; *μ*=3; *δ*=9; *E* = [−5, +5]; *Q* = {1,2,3,4,5,6};

**Table 6:** Sentiment classification on Android, Automotive, Electronic, and Movie datasets.

Android									
Metric\ model	RND	AFINN	RND + AFINN	JST	TJST	RJST	TS	WJST	WJST1
Accuracy1	0.48	0.6975	0.58	0.625	0.765	0.5825	0.5425	0.795	0.865
Accuracy2	—	—	—	—	—	—	—	0.7825	0.8525
Perplexity	—	—	—	17.4581	19.7185	17.4426	17.8706	14.396	14.7631
Topic_coherency	—	—	—	−2.0645	−0.8536	−1.9914	−2.373	−0.5547	−0.187

*Automotive*									
Accuracy1	0.4925	0.625	0.535	0.6575	0.7675	0.615	0.5525	0.755	0.8
Accuracy2	—	—	—	—	—	—	—	0.7475	0.795
Perplexity	—	—	—	22.6838	24.0385	21.8044	22.4878	18.4612	19.0627
Topic_coherency	—	—	—	−1.0158	−0.4712	−1.4986	−0.9008	−0.9311	−0.326

*Electronic*									
Accuracy1	0.465	0.675	0.52	0.7025	0.76	0.5525	0.5475	0.8625	0.8475
Accuracy2	—	—	—	—	—	—	—	0.875	0.855
Perplexity	—	—	—	23.3586	24.3024	23.471	24.0239	19.2999	20.2452
Topic_coherency	—	—	—	−1.5892	−1.0482	−1.2996	−1.2719	−0.5322	−1.1683

*Movie*									
Accuracy1	0.525	0.595	0.555	0.7575	0.9475	0.62	0.5425	0.8475	0.97
Accuracy2	—	—	—	—	—	—	—	0.8325	0.9675
Perplexity	—	—	—	25.2787	26.5813	25.1684	25.4488	21.0494	22.1082
Topic_coherency	—	—	—	−0.4089	−0.111	−1.0947	−1.0214	−0.0602	−0.1329

**Table 7:** Sentiment classification on Magazine, Sport, MR, Amazon, IMDB, and Yelp datasets.

Magazine									
Metric\ model	RND	AFINN	RND + AFINN	JST	TJST	RJST	TS	WJST	WJST1
Accuracy1	0.515	0.6522	0.5822	0.6705	0.705	0.5411	0.5022	0.8355	0.81
Accuracy2	—	—	—	—	—	—	—	0.8372	0.8083
Perplexity	—	—	—	21.8506	23.0349	21.4593	21.3828	19.9914	21.2095
Topic_coherency	—	—	—	−0.0548	−0.0348	−0.0946	−0.0561	−0.132	−0.0077

*Sport*									
Accuracy1	0.5285	0.686	0.5725	0.653	0.709	0.5565	0.5155	0.798	0.802
Accuracy2	—	—	—	—	—	—	—	0.782	0.795
Perplexity	—	—	—	22.874	23.1356	22.0264	22.3361	21.968	21.4821
Topic_coherency	—	—	—	−0.2234	−0.0876	−0.1369	−0.0544	−0.1406	−0.2242

*MR*									
Accuracy1	0.4895	0.601	0.5455	0.613	0.62	0.51	0.5	0.821	0.8445
Accuracy2	—	—	—	—	—	—	—	0.818	0.843
Perplexity	—	—	—	33.8663	35.0695	35.2359	34.6704	33.222	33.7698
Topic_coherency	—	—	—	−0.021	−0.0139	−0.0012	−0.0409	−0.001	−0.0106

*Amazon*									
Accuracy1	0.491	0.731	0.574	0.611	0.645	0.609	0.54	0.779	0.796
Accuracy2	—	—	—	—	—	—	—	0.829	0.798
Perplexity	—	—	—	12.6442	13.7316	13.349	14.2397	10.9211	12.9946
Topic_coherency	—	—	—	−0.8318	−4.3775	−0.4224	−0.5411	−0.5874	−0.2696

*IMDB*									
Accuracy1	0.498	0.698	0.575	0.605	0.616	0.546	0.545	0.76	0.77
Accuracy2	—	—	—	—	—	—	—	0.761	0.774
Perplexity	—	—	—	21.1053	20.4419	20.8543	19.7124	14.6719	18.9035
Topic_coherency	—	—	—	−1.3334	−1.4868	−0.8853	−1.1246	−0.9666	−0.9438

*Yelp*									
Accuracy1	0.506	0.689	0.559	0.579	0.614	0.561	0.547	0.737	0.773
Accuracy2	—	—	—	—	—	—	—	0.726	0.769
Perplexity	—	—	—	15.4565	16.7169	15.5965	15.3614	12.2145	13.3453
Topic_coherency	—	—	—	−2.1865	−2.5609	−1.9632	−1.1714	−2.0815	−2.3715

**Table 8:** Sentiment classification on different datasets based on different situations (AFINN and NO_AFINN).

Android			
Metric	Metric\Dic	AFINN	NO_AFINN
WJST	Accuracy1	0.7425	0.5725
	Accuracy2	0.7375	0.58
	Perplexity	15.53	16.1551
	Topic_Coh	−2.2654	−1.7346
WJST1	Accuracy1	0.855	0.81
	Accuracy2	0.8475	0.8075
	Perplexity	16.3399	16.0482
	Topic_Coh	−2.2228	−0.1295

*Automotive*			
WJST	Accuracy1	0.7125	0.6025
	Accuracy2	0.7025	0.6075
	Perplexity	20.4488	20.4065
	Topic_Coh	−3.2282	−1.6628
WJST1	Accuracy1	0.7925	0.7025
	Accuracy2	0.79	0.7125
	Perplexity	20.5213	21.1296
	Topic_Coh	−0.326	−1.1809

*Electronic*			
WJST	Accuracy1	0.8525	0.705
	Accuracy2	0.8425	0.6825
	Perplexity	20.0579	20.2615
	Topic_Coh	−0.5322	−0.5926
WJST1	Accuracy1	0.8475	0.76
	Accuracy2	0.855	0.765
	Perplexity	21.8195	21.5739
	Topic_Coh	−1.5586	−1.6968

*Movie*			
WJST	Accuracy1	0.8475	0.71
	Accuracy2	0.8325	0.715
	Perplexity	22.4588	22.6124
	Topic_Coh	−0.9342	−0.4637
WJST1	Accuracy1	0.9575	0.485
	Accuracy2	0.945	0.4875
	Perplexity	23.5662	22.8134
	Topic_Coh	−1.2359	−1.3717

**Table 9:** Sentiment classification on the Android dataset according to the different number of topics.

Model	Metric\topic	5	10	15	20
RND	Accuracy	0.48	0.48	0.48	0.48
AFINN	Accuracy	0.6975	0.6975	0.6975	0.6975
AFINN + RND	Accuracy	0.58	0.58	0.58	0.58
Bing_Liu	Accuracy	0.6975	0.6975	0.6975	0.6975
Bing_Liu + RND	Accuracy	0.5775	0.5775	0.5775	0.5775
IMDB	Accuracy	0.7025	0.7025	0.7025	0.7025
IMDB + RND	Accuracy	0.6125	0.6125	0.6125	0.6125
8K	Accuracy	0.5425	0.5425	0.5425	0.5425
8K + RND	Accuracy	0.515	0.515	0.515	0.515
JST	Accuracy	0.625	0.6175	0.6225	0.6125
	Perplexity	19.6726	19.5187	19.182	17.4581
	Topic_Coh	−4.6026	−2.5848	−2.2753	−2.0645
TJST	Accuracy	0.7575	0.7175	0.765	0.7475
	Perplexity	21.1487	20.3726	20.1516	19.7185
	Topic_Coh	−0.8536	−1.568	−3.4285	−2.8739
RJST	Accuracy	0.5825	0.54	0.555	0.5325
	Perplexity	19.9429	19.1915	18.137	17.4426
	Topic_Coh	−3.3792	−1.9914	−3.403	−3.4386
TS	Accuracy	0.5425	0.5275	0.53	0.5175
	Perplexity	20.4934	19.1762	18.3833	17.8706
	Topic_Coh	−3.9618	−3.2137	−2.373	−2.569
WJST	Accuracy1	0.7925	0.7425	0.795	0.79
	Accuracy2	0.7775	0.7375	0.775	0.7825
	Perplexity	16.7303	15.53	14.6351	14.396
	Topic_Coh	−0.5547	−2.2654	−2.9122	−2.6465
WJST1	Accuracy1	0.81	0.855	0.865	0.85
	Accuracy2	0.7925	0.8475	0.8525	0.8375
	Perplexity	16.6787	16.3399	15.6662	14.7631
	Topic_Coh	−0.187	−2.2228	−1.5703	−2.0204

**Table 10:** Sentiment classification on the Automotive dataset according to the different number of topics.

Model	Metric\topic	5	10	15	20
RND	Accuracy	0.4925	0.4925	0.4925	0.4925
AFINN	Accuracy	0.625	0.625	0.625	0.625
AFINN + RND	Accuracy	0.535	0.535	0.535	0.535
Bing_Liu	Accuracy	0.64	0.64	0.64	0.64
Bing_Liu + RND	Accuracy	0.535	0.535	0.535	0.535
IMDB	Accuracy	0.59	0.59	0.59	0.59
IMDB + RND	Accuracy	0.4825	0.4825	0.4825	0.4825
8K	Accuracy	0.5025	0.5025	0.5025	0.5025
8K + RND	Accuracy	0.48	0.48	0.48	0.48
JST	Accuracy	0.6275	0.6575	0.6325	0.5975
	Perplexity	24.7154	23.6961	23.27	22.6838
	Topic_Coh	−1.486	−2.3443	−1.1354	−1.0158
TJST	Accuracy	0.76	0.7375	0.7675	0.7275
	Perplexity	25.5637	25.0633	24.5749	24.0385
	Topic_Coh	−0.59	−0.4712	−1.6252	−1.9377
RJST	Accuracy	0.615	0.55	0.54	0.535
	Perplexity	25.2654	23.8316	22.3905	21.8044
	Topic_Coh	−1.5779	−1.4986	−2.0833	−2.9936
TS	Accuracy	0.5425	0.5375	0.5525	0.55
	Perplexity	25.0705	23.8504	22.9364	22.4878
	Topic_Coh	−2.5941	−0.9008	−6.2652	−4.6902
WJST	Accuracy1	0.755	0.7125	0.74	0.745
	Accuracy2	0.7475	0.7025	0.745	0.735
	Perplexity	21.2008	20.4488	18.7049	18.4612
	Topic_Coh	−1.6542	−3.2282	−1.6783	−0.9311
WJST1	Accuracy1	0.80	0.7925	0.7925	0.7925
	Accuracy2	0.79	0.79	0.79	0.795
	Perplexity	21.4357	20.5213	20.0481	19.0627
	Topic_Coh	−1.1883	−0.326	−1.084	−0.8349

**Table 11:** Sentiment classification on the Electronic dataset according to the different number of topics.

Model	Metric\topic	5	10	15	20
RND	Accuracy	0.465	0.465	0.465	0.465
AFINN	Accuracy	0.675	0.675	0.675	0.675
AFINN + RND	Accuracy	0.52	0.52	0.52	0.52
Bing_Liu	Accuracy	0.695	0.695	0.695	0.695
Bing_Liu + RND	Accuracy	0.535	0.535	0.535	0.535
IMDB	Accuracy	0.6375	0.6375	0.6375	0.6375
IMDB + RND	Accuracy	0.5475	0.5475	0.5475	0.5475
8K	Accuracy	0.5075	0.5075	0.5075	0.5075
8K + RND	Accuracy	0.5075	0.5075	0.5075	0.5075
JST	Accuracy	0.675	0.7025	0.6475	0.6275
	Perplexity	25.227	24.6719	24.6115	23.3586
	Topic_Coh	−1.5892	−4.1751	−2.6007	−2.1239
TJST	Accuracy	0.75	0.74	0.73	0.76
	Perplexity	25.2091	24.8028	24.4092	24.3024
	Topic_Coh	−1.0482	−1.8084	−1.6694	−1.8297
RJST	Accuracy	0.5525	0.55	0.5375	0.53
	Perplexity	25.3113	24.1779	23.8378	23.471
	Topic_Coh	−1.2996	−1.3991	−4.472	−5.4363
TS	Accuracy	0.54	0.5375	0.5475	0.515
	Perplexity	26.2295	24.885	24.5048	24.0239
	Topic_Coh	−1.2719	−1.6586	−2.5403	−3.2174
WJST	Accuracy1	0.8625	0.8525	0.7675	0.675
	Accuracy2	0.875	0.8425	0.755	0.665
	Perplexity	20.5489	20.0579	19.9009	19.2999
	Topic_Coh	−2.0442	−0.5322	−1.3702	−1.0887
WJST1	Accuracy1	0.79	0.8475	0.8075	0.8275
	Accuracy2	0.80	0.855	0.8125	0.8225
	Perplexity	22.6012	21.8195	20.8551	20.2452
	Topic_Coh	−1.1683	−1.5586	−1.6646	−1.4681

**Table 12:** Sentiment classification on the Movie dataset according to the different number of topics.

Model	Metric\topic	5	10	15	20
RND	Accuracy	0.525	0.525	0.525	0.525
AFINN	Accuracy	0.595	0.595	0.595	0.595
AFINN + RND	Accuracy	0.555	0.555	0.555	0.555
Bing_Liu	Accuracy	0.635	0.635	0.635	0.635
Bing_Liu + RND	Accuracy	0.565	0.565	0.565	0.565
IMDB	Accuracy	0.6425	0.6425	0.6425	0.6425
IMDB + RND	Accuracy	0.5975	0.5975	0.5975	0.5975
8K	Accuracy	0.5025	0.5025	0.5025	0.5025
8K + RND	Accuracy	0.51	0.51	0.51	0.51
JST	Accuracy	0.7575	0.6375	0.7175	0.6325
	Perplexity	27.3111	26.9145	26.2463	25.2787
	Topic_Coh	−1.0123	−0.4089	−1.2434	−1.226
TJST	Accuracy	0.915	0.9425	0.9475	0.9325
	Perplexity	27.791	27.4122	26.993	26.5813
	Topic_Coh	−0.111	−1.6632	−0.199	−0.8503
RJST	Accuracy	0.62	0.54	0.5175	0.5175
	Perplexity	27.6284	26.5172	26.1489	25.1684
	Topic_Coh	−2.6713	−1.0947	−1.979	−2.3544
TS	Accuracy	0.5425	0.515	0.5175	0.5175
	Perplexity	27.6707	26.4786	26.1138	25.4488
	Topic_Coh	−1.5025	−1.3799	−1.0214	−4.6598
WJST	Accuracy1	0.7225	0.8475	0.7525	0.6975
	Accuracy2	0.71	0.8325	0.7575	0.6875
	Perplexity	23.1145	22.4588	21.2982	21.0494
	Topic_Coh	−0.0751	−0.9342	−0.0602	−0.4873
WJST1	Accuracy1	0.97	0.9575	0.9675	0.9675
	Accuracy2	0.9625	0.945	0.9675	0.96
	Perplexity	24.6302	23.5662	22.7180	22.1082
	Topic_Coh	−0.1348	−1.2359	−0.1329	−0.7102

**Table 13:** Sentiment classification on different datasets according to the different number of distinct windows (before random selection).

Android							
Model	Metric\window	1	2	3	4	5	6
WJST	Accuracy1	0.8375	0.78	0.7925	0.7425	0.725	0.9075
	Accuracy2	0.83	0.77	0.7775	0.735	0.725	0.9075
	Perplexity	18.3948	16.7266	16.7303	16.166	15.9499	15.1128
	Topic_Coh	−1.4063	−0.9279	−0.5547	−1.2929	−1.7475	−0.746
WJST1	Accuracy1	0.8975	0.8525	0.81	0.8675	0.755	0.8025
	Accuracy2	0.8825	0.83	0.7925	0.8675	0.75	0.7875
	Perplexity	19.3734	18.0031	16.6787	16.8518	16.1015	16.5461
	Topic_Coh	−2.2084	−1.6272	−0.1870	−0.7753	−1.328	−1.7836

*Automotive*							
WJST	Accuracy1	0.7775	0.74	0.755	0.735	0.7375	0.69
	Accuracy2	0.7725	0.745	0.7475	0.745	0.725	0.685
	Perplexity	23.6365	21.7712	21.2008	20.9688	20.5095	19.6748
	Topic_Coh	−1.1684	−1.7095	−1.6542	−0.2824	−1.7604	−0.1311
WJST1	Accuracy1	0.805	0.8125	0.8	0.7575	0.78	0.7725
	Accuracy2	0.805	0.7975	0.79	0.755	0.78	0.7725
	Perplexity	23.2684	22.2601	21.4357	20.8092	20.5091	20.2379
	Topic_Coh	−1.7318	−0.5912	−1.1883	−0.7637	−0.62	−0.5134

*Electronic*							
WJST	Accuracy1	0.845	0.7675	0.8625	0.7325	0.7775	0.7325
	Accuracy2	0.845	0.7575	0.875	0.7275	0.7825	0.7225
	Perplexity	22.561	22.0462	20.5489	20.7952	20.7495	20.0283
	Topic_Coh	−0.8471	−0.6207	−2.0442	−1.4345	−0.786	−0.9412
WJST1	Accuracy1	0.8025	0.875	0.79	0.8675	0.8625	0.835
	Accuracy2	0.7975	0.87	0.8	0.865	0.8625	0.8375
	Perplexity	23.5903	23.546	22.6012	22.8559	21.8897	21.4464
	Topic_Coh	−0.8251	−0.9581	−1.1683	−1.2189	−0.8492	−0.3402

*Movie*							
WJST	Accuracy1	0.855	0.815	0.7225	0.6575	0.7	0.765
	Accuracy2	0.845	0.7975	0.71	0.6625	0.685	0.765
	Perplexity	24.5779	24.5153	23.1145	23.1209	23.2709	22.2328
	Topic_Coh	−0.4063	−0.0216	−0.0751	−2.5351	−0.0888	−0.0791
WJST1	Accuracy1	0.9725	0.9775	0.97	0.965	0.575	0.595
	Accuracy2	0.96	0.965	0.9625	0.955	0.5875	0.5725
	Perplexity	26.117	25.09	24.6302	23.9778	22.7068	22.3572
	Topic_Coh	−0.1348	−0.1348	−0.1348	−0.0315	−0.0378	−0.0106

*Average section*							
WJST	Accuracy1	0.8287	0.7756	0.7831	0.7168	0.735	0.7737
	Accuracy2	0.8231	0.7675	0.7775	0.7175	0.7293	0.77
	Perplexity	22.2925	21.2648	20.3986	20.2627	20.1199	19.2621
	Topic_Coh	−0.957	−0.8199	−1.082	−1.3862	−1.0956	−0.4743
WJST1	Accuracy1	0.8693	0.8793	0.8425	0.8643	0.7431	0.7512
	Accuracy2	0.8612	0.8656	0.8362	0.8606	0.745	0.7425
	Perplexity	23.0872	22.2248	21.3364	21.1236	20.3017	20.1469
	Topic_Coh	−1.225	−0.8278	−0.6696	−0.6973	−0.7087	−0.6619

**Table 14:** Sentiment classification on different datasets according to the different number of distinct windows (after random selection).

Android							
Model	Metric\window	1	2	3	4	5	6
WJST	Accuracy1	0.71	0.775	0.7025	0.69	0.7175	0.675
	Accuracy2	0.7	0.765	0.6975	0.68	0.705	0.6675
	Perplexity	17.791	15.8544	16.1124	15.6932	13.8904	14.1876
	Topic_Coh	−1.6	−1.9691	−1.4526	−4.6888	−2.6353	−1.2267
WJST1	Accuracy1	0.8025	0.85	0.845	0.8025	0.8675	0.76
	Accuracy2	0.7925	0.83	0.84	0.7875	0.865	0.7525
	Perplexity	18.9319	17.7849	17.6145	15.8017	15.4004	15.0917
	Topic_Coh	−1.2666	−1.6744	−3.027	−2.3428	−1.1215	−2.0183

*Automotive*							
WJST	Accuracy1	0.7425	0.7375	0.68	0.6825	0.6725	0.64
	Accuracy2	0.74	0.73	0.6775	0.68	0.6625	0.6325
	Perplexity	20.8433	20.7212	19.6843	20.0801	19.1691	18.7129
	Topic_Coh	−1.4406	−2.0489	−2.6701	−2.2155	−1.1019	−1.1651
WJST1	Accuracy1	0.7825	0.76	0.755	0.7725	0.7475	0.7675
	Accuracy2	0.785	0.7575	0.7475	0.775	0.7525	0.76
	Perplexity	22.1856	21.3601	20.6057	19.8915	19.402	19.0827
	Topic_Coh	−1.5638	−1.474	−1.1274	−1.7761	−1.2042	−1.1755

*Electronic*							
WJST	Accuracy1	0.7975	0.72	0.76	0.6875	0.7675	0.6825
	Accuracy2	0.795	0.715	0.7475	0.66	0.765	0.665
	Perplexity	21.9171	21.0509	20.4858	20.26	19.4089	19.5549
	Topic_Coh	−0.8282	−1.0407	−0.9593	−0.9779	−1.0413	−0.6259
WJST1	Accuracy1	0.7475	0.7425	0.7775	0.7425	0.7725	0.78
	Accuracy2	0.745	0.7475	0.78	0.74	0.7625	0.785
	Perplexity	23.2269	22.4573	21.7203	21.4598	20.8683	20.5084
	Topic_Coh	−1.7905	−1.5082	−1.4854	−0.9143	−1.2881	−1.3489

*Movie*							
WJST	Accuracy1	0.7425	0.77	0.8075	0.615	0.7	0.595
	Accuracy2	0.7425	0.765	0.7875	0.59	0.69	0.595
	Perplexity	23.7529	23.322	22.1056	21.8573	20.8467	20.9653
	Topic_Coh	−1.0908	−0.9134	−0.9145	−0.6129	−1.1099	−0.716
WJST1	Accuracy1	0.9725	0.965	0.96	0.9675	0.96	0.9625
	Accuracy2	0.975	0.96	0.955	0.955	0.9575	0.9575
	Perplexity	26.1797	25.3138	24.5149	24.1116	23.5025	23.3023
	Topic_Coh	−0.0218	−0.2225	−1.9033	−0.0897	−0.2896	−0.0569

*Average section*							
WJST	Accuracy1	0.7481	0.7506	0.7375	0.6687	0.7143	0.6481
	Accuracy2	0.7443	0.7437	0.7275	0.6525	0.7056	0.64
	Perplexity	21.076	20.2371	19.597	19.4726	18.3287	18.3551
	Topic_Coh	−1.2399	−1.493	−1.4991	−2.1237	−1.4721	−0.9334
WJST1	Accuracy1	0.8262	0.8293	0.8343	0.8212	0.8368	0.8175
	Accuracy2	0.8243	0.8237	0.8306	0.8143	0.8343	0.8137
	Perplexity	22.631	21.729	21.1138	20.3161	19.7933	19.4962
	Topic_Coh	−1.1606	−1.2197	−1.8857	−1.2807	−0.9758	−1.1499

**Table 15:** Sentiment scores, for some instance, words related to Android, Automotive, Electronic, and Movie datasets.

Dataset	Android		Automotive		Electronic		Movie	
Model	Word	Score	Word	Score	Word	Score	Word	Score
*WJST1*	Nice	5	Much	5	Satisfy	5	See	5
	Cute	4	Use	4	Crew	4	Father	4
	Favorit	3	Long	3	Way	3	Pray	3
	Perfect	2	Expens	2	Fluid	2	Human	2
	Great	1	Stuff	1	Feel	1	Event	1
	Type	−1	Fals	−1	Pull	−1	Terribl	−1
	Wast	−2	Serious	−2	Side	−2	Sens	−2
	Everi	−3	Extens	−3	Nervous	−3	Lost	−3
	Unknown	−4	Space	−4	Even	−4	Sure	−4
	Everyth	−5	Know	−5	Extend	−5	Injur	−5

*WJST*	Nice	4	Much	3	Satisfy	−2	See	−4
	Cute	1	Use	2	Crew	2	Father	5
	Favorit	2	Long	4	Way	4	Pray	−3
	Perfect	2	Expens	−4	Fluid	1	Human	5
	Great	2	Stuff	−5	Feel	−3	Event	1
	Type	1	Fals	−5	Pull	−4	Terribl	4
	Wast	−5	Serious	1	Side	−1	Sens	−4
	Everi	4	Extens	5	Nervous	3	Lost	3
	Unknown	5	Space	−5	Even	−2	Sure	2
	Everyth	2	Know	4	Extend	−5	Injur	−5

**Table 16:** Sentiment scores for some instance words related to STS, Sanders, and SOMD datasets.

Dataset		STS	Sanders	SOMD
Model	Word	Score	Score	Score
WJST1	Much	−4	4	2
	Good	5	4	−5
	Bad	−5	−1	−5
	Nice	−3	5	5
	Hate	−5	−5	1
	Love	5	5	3

WJST	Much	−5	−5	−2
	Good	−4	−1	3
	Bad	−4	−1	3
	Nice	3	2	1
	Hate	−5	−4	2
	Love	4	−2	−3

**Table 17:** Top 10 words extracted from the Movie dataset.

Model	Sentiment	Top 10 words
WJST	**+**	jesu, film, God, love, mel, Christian, life, suffer, believ, roman
	**−**	movi, godzilla, bad, dvd, origin, horror, buy, version, worst, actor

WJST1	**+**	Jesu, mel, passion, mother, stori, realli, great, everyon, God, like
	**−**	godzilla, monster, go, time, star, know, kill, make, militari, American

JST	**+**	mel, stori, mother, two, realli, becom, anoth, God, like, back
	**−**	godzilla, look, monster, american, militari, like, worst, zellweg, emmerich, quit

**Table 18:** Top 10 words extracted from Android dataset.

Model	Sentiment	Top 10 words
WJST	**+**	app, game, sudoku, play, version, enjoy, option, want, hint, like
	**−**	work, app, would, fire, live, station, tri, say, select, kindl, load, user

WJST1	**+**	sudoku, tri, love, game, time, easi, tablet, star, call, make
	**−**	close, tablet, seem, get, year, download, much, station, time, android

JST	**+**	station, want, even, peopl, work, avail, version, puzzl, custom, believ
	**−**	use, app, find, review, great, got, total, new, night, fake

**Table 19:** Top 10 words extracted from Electronic dataset.

Model	Sentiment	Top 10 words
WJST	**+**	read, book, screen, touch, kindl, page, better, wifi, ebook, like
	**−**	work, went, new, servic, need, bad, system, hous, number, mine

WJST1	**+**	googl, amazon, book, color, store, kindl, download, small, pdf
	**−**	time, work, two, much, one, power, comput, phone, go, unit

JST	**+**	book, touch, read, page, free, librari, touch, screen, much, pdf
	**−**	plug, work, could, devic, comput, charger, router, cabl, item, design

**Table 20:** Sentiment classification using different sentiment dictionaries achieved by WJST.

Model\dataset	Android	Auto	ELEC	MOV	STS	Sanders	SOMD
RND	0.48	0.4925	0.465	0.525	0.5346	0.4852	0.4847
AFINN	0.6975	0.625	0.675	0.595	0.734	0.674	0.3395
AFINN + w	0.685	0.58	0.6375	0.595	—	—	—
AFINN + RND	0.58	0.535	0.52	0.555	0.6038	0.5424	0.4475
Bing_Liu	0.6975	0.64	0.695	0.635	0.698	0.6813	0.322
Bing_Liu + RND	0.5775	0.535	0.535	0.565	0.6288	0.5416	0.4388
IMDB	0.7025	0.59	0.6375	0.6425	0.5512	0.5988	0.4617
IMDB + RND	0.6125	0.4825	0.5475	0.5975	0.5595	0.5196	0.4748
8K	0.5425	0.5025	0.5075	0.5025	0.4986	0.5351	0.3995
8K + RND	0.515	0.48	0.5075	0.51	0.5373	0.495	0.4814
Android	0.8525	0.62	0.645	0.6125	0.6023	0.6078	0.4712
Android + w	0.8375	0.59	0.63	0.62	—	—	—
Android + RND	0.8525	0.6275	0.6675	0.61	0.5995	0.5865	0.4832
Auto	0.57	0.705	0.6375	0.605	0.6023	0.5767	0.6011
Auto + w	0.5775	0.7	0.65	0.6075	—	—	—
Auto + RND	0.5625	0.705	0.6175	0.5875	0.6106	0.5522	0.588
ELEC	0.6525	0.5975	0.8425	0.4925	0.6023	0.6029	0.5323
ELEC + w	0.6625	0.605	0.8275	0.5025	—	—	—
ELEC + RND	0.645	0.6075	0.8425	0.4775	0.6023	0.6037	0.5585
MOV	0.66	0.5925	0.6375	0.81	0.6244	0.535	0.5127
MOV + w	0.6725	0.59	0.6475	0.8025	—	—	—
MOV + RND	0.6525	0.5875	0.62	0.81	0.6217	0.5416	0.5105
STS	0.51	0.58	0.575	0.615	0.624	0.4762	0.5312
STS + RND	0.5575	0.5625	0.5275	0.5775	0.624	0.5138	0.54
Sanders	0.59	0.6	0.565	0.5575	0.5607	0.7088	0.5105
Sanders + RND	0.5925	0.5925	0.5825	0.5425	0.5746	0.7088	0.5443
SOMD	0.5525	0.5275	0.47	0.4625	0.4859	0.5334	0.6093
SOMD + RND	0.5375	0.5	0.4875	0.4725	0.5441	0.5236	0.6093

**Table 21:** Sentiment classification using different sentiment dictionaries achieved by WJST1.

Model\dataset	Android	Auto	ELEC	MOV	STS	Sanders	SOMD
RND	0.48	0.4925	0.465	0.525	0.5346	0.4852	0.4847
AFINN	0.6975	0.625	0.675	0.595	0.734	0.674	0.3395
AFINN + w	0.685	0.58	0.6375	0.595	—	—	—
AFINN + RND	0.58	0.535	0.52	0.555	0.6038	0.5424	0.4475
Bing_Liu	0.6975	0.64	0.695	0.635	0.698	0.6813	0.322
Bing_Liu + RND	0.5775	0.535	0.535	0.565	0.6288	0.5416	0.4388
IMDB	0.7025	0.59	0.6375	0.6425	0.5512	0.5988	0.4617
IMDB + RND	0.6125	0.4825	0.5475	0.5975	0.5595	0.5196	0.4748
8K	0.5425	0.5025	0.5075	0.5025	0.4986	0.5351	0.3995
8K + RND	0.515	0.48	0.5075	0.51	0.5373	0.495	0.4814
Android	0.855	0.6025	0.565	0.65	0.5857	0.5865	0.4985
Android + w	0.835	0.6	0.6025	0.6625	—	—	—
Android + RND	0.855	0.59	0.575	0.6125	0.5967	0.5579	0.505
Auto	0.6375	0.745	0.6175	0.63	0.594	0.6233	0.4766
Auto + w	0.6325	0.7375	0.65	0.635	—	—	—
Auto + RND	0.6375	0.745	0.62	0.6225	0.5829	0.6118	0.4875
ELEC	0.65	0.5475	0.7075	0.5825	0.594	0.6192	0.457
ELEC + w	0.63	0.5775	0.7125	0.63	—	—	—
ELEC + RND	0.6425	0.5575	0.7075	0.5775	0.6134	0.5914	0.4744
MOV	0.63	0.5525	0.5825	0.6375	0.5663	0.5767	0.4603
MOV + w	0.6425	0.5475	0.6425	0.94	—	—	—
MOV + RND	0.635	0.565	0.61	0.6375	0.5718	0.571	0.4493
STS	0.585	0.5475	0.595	0.5775	0.7431	0.5939	0.4231
STS + RND	0.5875	0.565	0.5975	0.5725	0.7431	0.5743	0.4395
Sanders	0.605	0.555	0.6	0.5375	0.6632	0.7538	0.421
Sanders + RND	0.6075	0.55	0.6025	0.5375	0.6771	0.7538	0.4559
SOMD	0.615	0.555	0.55	0.5125	0.5773	0.6078	0.594
SOMD + RND	0.61	0.5275	0.5425	0.5325	0.594	0.5767	0.594

**Table 22:** Description of the multidomain dataset used in this section.

#	Domains	Number of reviews	Vocabulary size	Number of words
1	Android, Automotive, Electronic, and Movie	1600	11183	100113

**Table 23:** Sentiment classification on MR, Sanders, SOMD, STS, Amazon, IMDB, and Yelp datasets.

Method/dataset	MR	Sanders	SOMD	STS	Amazon	IMDB	Yelp
ALGA [46]	—	0.8067	0.8147	0.7668	—	—	—
ALGA-SW [46]	—	0.7868	0.7877	0.7886	—	—	—
BPSO (Shang et al., 2016)	—	—	—	—	0.7439	0.79	0.789
BICA (Mirhosseini et al., 2017)	—	—	—	—	0.793	0.745	0.763
BABC (Schiezaro et al., 2013)	—	—	—	—	0.7509	0.74	0.736
MaxEnt (Saif et al., 2014)	—	0.8362	—	0.7782	—	—	—
NB (Saif et al., 2014)	—	0.8266	—	0.8106	—	—	—
LS-all [13]	—	0.8199	—	—	—	—	—
SVM-all [13]	—	0.8214	—	—	—	—	—
RMTL [13]	—	0.827875	—	—	—	—	—
MTL-graph [13]	—	0.801725	—	—	—	—	—
CMSC [13]	—	0.846325	—	—	—	—	—
LSTM-all [13]	—	0.8063	—	—	—	—	—
MTL-CNN [13]	—	0.829825	—	—	—	—	—
MTL-DNN [13]	—	0.817	—	—	—	—	—
ASP-MTL [13]	—	0.85125	—	—	—	—	—
NeuroSent [13]	—	0.834575	—	—	—	—	—
DAM [13]	—	0.863225	—	—	—	—	—
SVM-BoW (Da Silva et al., 2014)	—	0.8243	0.7402	—	—	—	—
SVM-BoW + lex (Da Silva et al., 2014)	—	0.8398	0.7893	—	—	—	—
RF-BoW (Da Silva et al., 2014)	—	0.7924	0.7391	—	—	—	—
RF-BoW + lex (Da Silva et al., 2014)	—	0.8235	0.7936	—	—	—	—
LR-BoW (Da Silva et al., 2014)	—	0.7745	0.7238	—	—	—	—
LR-BoW + lex (Da Silva et al., 2014)	—	0.7949	0.7806	—	—	—	—
MNB-BoW (Da Silva et al., 2014)	—	0.7982	0.7543	—	—	—	—
MNB-BoW + lex (Da Silva et al., 2014)	—	0.8341	0.8013	—	—	—	—
ENS(LR + RF + MNB)-BoW (Da Silva et al., 2014)	—	0.8276	0.7555	—	—	—	—
ENS(LR + RF + MNB)-BoW + lex (Da Silva et al., 2014)	—	0.8489	0.8035	—	—	—	—
SVM-FH (Da Silva et al., 2014)	—	0.4975	0.5131	—	—	—	—
SVM-FH + lex (Da Silva et al., 2014)	—	0.7500	0.6299	—	—	—	—
RF-FH (Da Silva et al., 2014)	—	0.5564	0.6136	—	—	—	—
RF-FH + lex (Da Silva et al., 2014)	—	0.7163	0.7260	—	—	—	—
LR-FH (Da Silva et al., 2014)	—	0.5694	0.6529	—	—	—	—
LR-FH + lex (Da Silva et al., 2014)	—	0.7598	0.7303	—	—	—	—
MNB-FH (Da Silva et al., 2014)	—	0.5425	0.6070	—	—	—	—
MNB-FH + lex (Da Silva et al., 2014)	—	0.7508	0.7139	—	—	—	—
ENS(LR + RF + MNB)-FH (Da Silva et al., 2014)	—	0.5784	0.6517	—	—	—	—
ENS(LR + RF + MNB)-FH + lex (Da Silva et al., 2014)	—	0.7663	0.7456	—	—	—	—
WS-TSWE' [76]	0.841	—	—	—	—	—	—
WS-TSWE [76]	0.824	—	—	—	—	—	—
TSWE-P [76]	0.726	—	—	—	—	—	—
TSWE + P [76]	0.782	—	—	—	—	—	—
JSTH [76]	0.681	—	—	—	—	—	—
HTSM [76]	0.796	—	—	—	—	—	—
SAE (Pagliardini et al., 2018)	0.861	—	—	—	—	—	—
ParagraphVec DBOW (Pagliardini et al., 2018)	0.763	—	—	—	—	—	—
ParagraphVec DM (Pagliardini et al., 2018)	0.764	—	—	—	—	—	—
IST (Pu et al., 2019)	0.827	—	—	—	—	—	—
UST (Pu et al., 2019)	0.832	—	—	—	—	—	—
UIST (Pu et al., 2019)	0.845	—	—	—	—	—	—
RND	0.4895	0.4852	0.4847	0.5346	0.491	0.498	0.506
AFINN	0.601	0.674	0.3395	0.734	0.731	0.698	0.689
AFINN + RND	0.5455	0.5424	0.4475	0.6038	0.574	0.575	0.559
JST [8]	0.613	0.6176	0.4934	0.6398	0.611	0.605	0.579
TJST [8]	0.62	0.5898	0.4639	0.6814	0.645	0.616	0.614
RJST [12]	0.51	0.6086	0.4967	0.6232	0.609	0.546	0.561
TS [9]	0.5	0.5612	0.5251	0.565	0.54	0.545	0.547
WJST-1	0.821	0.7268	.75786	0.7126	0.779	0.76	0.737
WJST-2	0.818	0.7203	0.75775	0.7181	0.829	0.761	0.726
WJST1-1	0.8445	0.832	0.8	0.8317	0.796	0.77	0.773
WJST1-2	0.843	0.8352	0.7859	0.8373	0.798	0.774	0.769

**Table 24:** Sentiment classification in comparison with discriminative models on different datasets.

Method\Dataset		Android	Automotive	Electronic	Movie
RND		0.48	0.4925	0.465	0.525
AFINN		0.6975	0.625	0.675	0.595
RND + AFINN		0.58	0.535	0.52	0.555
LOGISTIC REGRESSION(BOW)		0.53	0.52	0.5338	0.57
RANDOMFOREST(BOW)		0.69	0.65	0.60	0.6575
SVM(BOW)		0.49	0.51	0.54	0.5675
DECISIONTREE(BOW)		0.71	0.70	0.56	0.665
NAIVE_BAYES(BOW)		0.51	0.55	0.4712	0.555
KNEIGHBORS (*N* = 3, BOW)		0.55	0.57	0.55	0.5725
KNEIGHBORS (*N* = 4,BOW)		0.56	0.575	0.5915	0.585
KNEIGHBORS (*N* = 5,BOW)		0.57	0.5725	0.55	0.59
KNEIGHBORS (*N* = 6,BOW)		0.57	0.5675	0.56	0.61
LOGISTIC REGRESSION (TF-IDF)		0.61	0.63	0.83	0.59
RANDOMFOREST (TF-IDF)		0.575	0.6	0.7243	0.5225
SVM (TF-IDF)		0.5825	0.59	0.80	0.54
DECISIONTREE (TF-IDF)		0.55	0.57	0.7043	0.55
NAIVE_BAYES (TF-IDF)		0.58	0.60	0.7945	0.58
KNEIGHBORS (*N* = 3, TF-IDF)		0.65	0.63	0.53	0.7125
KNEIGHBORS (*N* = 4, TF-IDF)		0.65	0.63	0.56	0.7125
KNEIGHBORS (*N* = 5, TF-IDF)		0.65	0.63	0.51	0.7125
KNEIGHBORS (*N* = 6, TF-IDF)		0.65	0.5725	0.53	0.7125
JST		0.625	0.6575	0.7025	0.7575
ASUM		0.613	0.6322	0.71	0.772
TJST		0.765	0.7675	0.76	0.9475
RJST		0.5825	0.615	0.5525	0.62
TS		0.5425	0.5525	0.5475	0.5425
WJST	Accuracy1	0.795	0.755	0.8625	0.8475
	Accuracy2	0.7825	0.7475	**0.875**	0.8325
WJST1	Accuracy1	**0.865**	**0.8**	0.8475	**0.97**
	Accuracy2	0.8525	0.795	0.855	0.9675

**Table 25:** Sentiment classification according to extended features (bigrams and trigrams) on different datasets (single-domain).

Model	Gram	Metric\dataset	Android	Automotive	Electronic	Movie
*JST*	**U ** ^*∗*^	Accuracy	0.625	0.6275	0.675	0.7575
		Perplexity	19.6726	24.7154	25.227	27.3111
		Topic_Coh	−4.6026	−1.486	−1.5892	−1.0123
	**U** **+** **B**^*∗*^	Accuracy	0.655	0.7025	0.75	0.835
		Perplexity	66.1882	86.6897	81.3375	98.9201
		Topic_Coh	−2.4595	−1.0417	−0.9823	−0.4165
	**U** **+** **B** **+** **T**^*∗*^	Accuracy	0.6775	0.5825	0.7525	0.7275
		Perplexity	114.8812	158.7113	156.1383	190.8403
		Topic_Coh	−1.5776	−2.3915	−0.7030	−0.1288

*WJST*	**U**	Accuracy1	0.7925	0.755	0.8625	0.7225
		Accuracy2	0.7775	0.7475	0.875	0.71
		Perplexity	16.7303	21.2008	20.5489	23.1145
		Topic_Coh	−0.5547	−1.6542	−2.0442	−0.0751
	**U** **+** **B**	Accuracy1	0.6825	0.63	0.67	0.65
		Accuracy2	0.68	0.625	0.6675	0.6475
		Perplexity	36.5505	49.8584	48.2008	58.0838
		Topic_Coh	−1.3976	−4.0214	−1.2513	−0.0423
	**U** **+** **B** **+** **T**	Accuracy1	0.7325	0.6825	0.635	0.6475
		Accuracy2	0.7325	0.6825	0.615	0.6325
		Perplexity	52.9205	72.7629	76.2545	88.1860
		Topic_Coh	−1.3242	−1.5525	−1.2409	−2.5170

*WJST1*	**U**	Accuracy1	0.81	0.80	0.79	0.97
		Accuracy2	0.7925	0.79	0.80	0.9625
		Perplexity	16.6787	21.4357	22.6012	24.6302
		Topic_Coh	−0.187	−1.1883	−1.1683	−0.1348
	**U** **+** **B**	Accuracy1	0.7	0.7625	0.8575	0.935
		Accuracy2	0.7	0.7625	0.8525	0.935
		Perplexity	38.5603	49.5167	54.7041	62.3459
		Topic_Coh	−2.0962	−1.8607	−0.9744	−0.0617
	**U** **+** **B** **+** **T**	Accuracy1	0.83	0.7625	0.745	0.9475
		Accuracy2	0.8275	0.7675	0.745	0.9475
		Perplexity	55.5819	75.3624	77.6660	96.3675
		Topic_Coh	−1.3837	−0.1840	−0.9334	−0.0333

U^*∗*^: unigram. B^*∗*^: bigram. T^*∗*^: trigram.

## Data Availability

The datasets used during the current study are available from the corresponding author (a.osmani@qiau.ac.ir) upon reasonable request.
